# Vulnerability to climate change of a microendemic lizard species from the central Andes

**DOI:** 10.1038/s41598-021-91058-w

**Published:** 2021-06-02

**Authors:** A. Laspiur, J. C. Santos, S. M. Medina, J. E. Pizarro, E. A. Sanabria, B. Sinervo, N. R. Ibargüengoytía

**Affiliations:** 1 Instituto de Investigaciones en Biodiversidad y Medioambiente, Consejo Nacional de Investigaciones Científicas y Técnicas (INIBIOMA-CONICET), Quintral 1250, 8400 San Carlos de Bariloche, Argentina; 2 Department of Biological Sciences, St. John’s University, 8000 Utopia Parkway, Queens, NY USA; 3 Centro de Investigación Esquel de Montaña y Estepa Patagónica, Consejo Nacional de Investigaciones Científicas y Técnicas (CIEMEP-CONICET), 9200 Esquel, Chubut, Argentina; 4 Departamento de Biología, Facultad de Ciencias Exactas, Físicas y Naturales, Universidad Nacional de San Juan, Av. José I. de la Roza 590 (0) Rivadavia, 5402 San Juan, Argentina; 5 Instituto de Ciencias Básicas, Facultad de Filosofía Humanidades y Artes, Universidad Nacional de San Juan - Consejo Nacional de Investigaciones Científicas y Técnicas (CONICET), Av. José I. de la Roza 230 (O), 5400 Capital, San Juan, Argentina; 6 Department of Ecology and Evolutionary Biology, Coastal Sciences Building, University of California, 130 McAllister Way, Santa Cruz, CA 95065 USA

**Keywords:** Ecology, Zoology

## Abstract

Given the rapid loss of biodiversity as consequence of climate change, greater knowledge of ecophysiological and natural history traits are crucial to determine which environmental factors induce stress and drive the decline of threatened species. *Liolaemus montanezi* (Liolaemidae), a xeric-adapted lizard occurring only in a small geographic range in west-central Argentina, constitutes an excellent model for studies on the threats of climate change on such microendemic species. We describe field data on activity patterns, use of microhabitat, behavioral thermoregulation, and physiology to produce species distribution models (SDMs) based on climate and ecophysiological data. *Liolaemus montanezi* inhabits a thermally harsh environment which remarkably impacts their activity and thermoregulation. The species shows a daily bimodal pattern of activity and mostly occupies shaded microenvironments. Although the individuals thermoregulate at body temperatures below their thermal preference they avoid high-temperature microenvironments probably to avoid overheating. The population currently persists because of the important role of the habitat physiognomy and not because of niche tracking, seemingly prevented by major rivers that form boundaries of their geographic range. We found evidence of habitat opportunities in the current range and adjacent areas that will likely remain suitable to the year 2070, reinforcing the relevance of the river floodplain for the species’ avoidance of extinction.

## Introduction

The consequences of global climate change on plants and animals have attracted a vast research endeavor on ecology and physiology over the last two decades as the rapid planetary warming becomes evident^1–3^. One of their main goals is the evaluation of physiological profiles on species and projections of how different climatic scenarios, derived from the monitoring of the climatological fluctuations and anomalies over the past century, might affect biodiversity. These ecophysiological models serve to perform long-term predictions of future climate as the Earth’s atmosphere changes by rapid accumulation of greenhouse gases^4^. Global warming indirectly leads to the alteration of other climatological events that also affect organismal population dynamics, such as shifts in seasonal rainfall patterns^5,6^, decreases in daily thermal amplitudes in some regions^7^, and more frequent climate anomalies or extremes^8^. At the same time, new advances and faster computational technologies have made possible more accurate predictions about future impacts of climate change on diverse biota^9^.

Current forecasts denote a substantial loss of biodiversity at multiple scales due to alterations in population distribution patterns and the exacerbation of environmental stressors. Such fluctuations might be extreme and lead organisms to their physiological limits and many populations might experience declines in size and local extinctions^10–13^. Therefore, one of the critical challenges for biologists is to identify which species and populations are likely to be critically affected by warming-driven changes and in which particular habitats. These results would provide to resource managers and policymakers an understanding of why species or habitats are or will become vulnerable to climate change and allow the development of strategies for mitigation, rehabilitation or rescue before the extinction spiral leads such taxa to extirpation or extinction^14^.

Distribution and abundance of most species could be altered as consequence of climate change^15–18^. However, species with limited dispersal capacities (low vagility) or confined to specialized habitats within restricted distribution ranges are particularly vulnerable. Hence, when these habitats develop conditions at the limits of species’ environmental envelopes, this scenario might irreversibly lead to the extirpation of such populations. The consequences might be worse if species under study are microendemics and such environmental extremes might lead to their extinction^12,13,19^. To respond to and mitigate such threats, current research efforts are focused on the identification of which species and populations are most vulnerable, and which aspects of their physiology and ecology might increase their vulnerability and what can be done (e.g., human translocation of founders groups to future suitable habitats). The implementation of conservation and management efforts of such vulnerable species and their habitats are one of the main outcomes of physiological ecology research^11^.

Vertebrate ectotherms, due to their marked dependence on environmental temperature (T_a_), are particularly vulnerable to thermal fluctuations. These threats include heat waves that directly influence physiology and behavior of vulnerable taxa^20–22^ which affect their performance and fitness^23,24^. For example, body temperature (T_b_) fluctuations in lizards affect several physiological and self-maintenance cornerstones including their digestion, metabolism, growth^22,23^, reproduction, and susceptibility to diseases^25–27^. Likewise, environmental temperature also influences locomotion and the ability to escape from predators, social interactions, feeding and reproductive behaviors^27–32^. In consequence, lizards have developed a variety of mechanisms to maintain their homeostasis affected by drastic changes in T_b_ as consequence of environmental thermal fluctuations^33,34^.

For instance, maintaining T_b_ within an optimal range for physiological and behavioral activities, will depend on the supply and quality of microhabitats available for thermoregulation, and also depends on the effectiveness of the individuals to exploit available resources^35,36^. Lizards can behaviorally regulate their T_b_ by moving among appropriate microhabitats, modifying the activity periods^37–40^, and adopting different thermoregulatory strategies^39,41^. The individual flexibility of making these physiological and behavioral adjustments depends on variability and phenotypic plasticity^22,42^. Therefore, if individuals fail in their behavioral or physiological responses to face environmental constraints, they will respond by spatial shifts to track for environments similar to those they are adapted (niche tracking) or afford the risk of extirpation^43^. Such negative impacts are amplified in species with limited dispersal capacities or confined to specialized habitats with small distribution ranges. Overall, these taxa are particularly vulnerable to rapid habitat degradation and are of special concern for conservation efforts (e.g., How do we prioritize or triage populations for conservation efforts?).

Many lizard species and populations are currently at risk of extinction due to an accelerated increase in the Earth’s temperature, mainly because rising ambient temperatures (T_a_) that exceed organismal thermal tolerance margins^13,44^. This is particularly true in tropical lizards, whose populations already experience T_a_ values close to their physiological optimum^45^. When the individuals’ T_b_s are close, or higher than the optimal temperatures (T_opt_), this results in physiological stress and consequently reduces the individuals’ performance^13,46^. Warmer temperatures also imply faster metabolism, which enhance aging leading to shorter lifespans^47^. Thus, increasing temperatures could be beneficial the near term, but if they reduce longevity over the long term, populations can become prone to extinction^47,48^. For example, if the projected T_a_ can increase 1.2 °C to 4 °C by 2050, many lizard populations could increase their metabolic rates^49^ in detriment of their longevity^47^. Likewise, if climatic disturbances prompt shifts in the thermal environments, many lizards may reduce the proportion of time outside their shelters performing social, reproductive, feeding or basking activities (hours of activity, h_a_) and increase the time spent in shelters (hours of restriction, h_r_; *sensu* Sinervo et al.^13^) having a negative impact on growth rates, and phenology of populations, resulting in an enhanced risk of extinction^50,51^. This conclusion has been documented in Mexican lizards and Sinervo et al.^13^ predicted that many other species have higher probabilities of extinction when forced to have fewer hours of activity to reach their optimal body temperatures.

High mountain environments, such as the Andes, constitute areas of great interest for biodiversity conservation because of the coexistence of different spatial units of vegetation, ecotones and natural gradients of elevation^52,53^. In particular, the central-western Andes (Argentina) have pronounced effects on temperature and humidity where the Puna and Monte biogeographic regions converge to form a heterogeneous mosaic of deserts^54^. This geospatial configuration contains several microendemic species of lizards including several taxa of the megadiverse *Liolaemus*^55–57^. For example, *L. montanezi* is a small-sized lizard (< 67 mm snout-vent length) with a unique life history^58^. For instance, these animals are oviparous and microendemic to a hyper-arid desert area with a restricted distribution range in Argentina (San Juan Province). Given its rarity, *L. montanezi* is categorized as “insufficient knowledge” by Abdala et al.^59^, consistent with the criteria for “Deficient Data” taxa by IUCN Red List of Threatened Species^60^. Unfortunately, this categorization makes *L. montanezi* a low priority for conservation as consequence of the limited studies on this taxon. However, based on our recent field work, we considered that *L. montanezi* population could be at an extreme risk of extinction by a combination of several factors. First, the geographical features of the species’ distribution are limited to a minute area of ~ 2 km^2^ limited by Blanco River to the east and its confluence with La Palca River to the southeast, and surrounded by the slopes of the Andes on the west and northwest sides. This reduced and confined habitat suggests an unsurpassable geographic restriction to overcome whenever habitat becomes too detrimental due to climate change for this species to shift in its distribution to a more suitable habitat. Secondly, the climate trends with strong warming and a substantial decrease of the precipitation during the period 1960 ‒ 2010 had impacted the biota in central-western Argentina which might have reduced the availability of shelter and food for *L. montanezi*^61^. Third, climate projections for this region based in long-term atmospheric conditions under RCP 4.5 and RCP 8.5 scenarios show a substantial increase of the temperature in the range of 2.0‒2.5 °C and 3.5 °C, respectively; and a drastic reduction of precipitation in northwestern Argentina by the year 2100^61^. Such climatic projections suggest an accentuated thermal and hydric constraint imposed by the environment as they challenge the persistence of the current *L. montanezi* population.

We hypothesize that the long-term persistence of *L. montanezi* could be threatened by climate change considering its microendemic condition and the present thermal, humidity, and dispersion restraints of its natural habitat. Herein, we analyze its current thermal niche, use of microhabitat, and activity patterns to predict *L. montanezi* warming tolerance, thermal safety margins, and the potential hours of restriction of activity. Using ecophysiological, geographic, and climatic parameters in Species Distribution Models (SDM) we predict probabilities of persistence, dispersion, or extirpation of this narrowly distributed population under global warming 2050 and 2070 scenarios^62^. Finally, we propose strategies for present and future conservation of *L. montanezi.*

## Results

### Relationships of T_b_, T_pref,_ VT_max_, CT_max_, CT_min_ with body size (SVL), body mass (BM) and body condition index (M_i_)

T_b_ and T_pref_ of *L. montanezi* were not related with SVL, BM or M_i_ (Supplementary Table S1). However, CT_max_ was negatively related to BM and SVL, and VT_max_ was positively related to M_i_ (Supplementary Table S1).

### Determination of the main thermal sources for thermoregulation, and relationship among T_b_ and climatic factors: wind velocity (Wd) and relative humidity (RH%)

**Figure 1:**
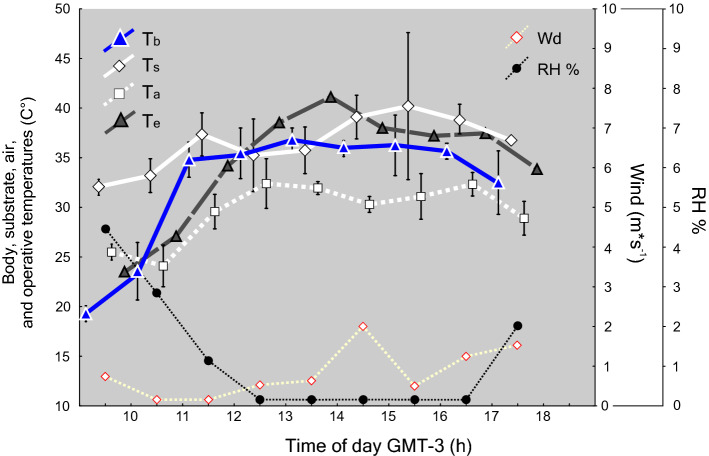
Variation during activity of mean body temperature (°C; T_b_, blue triangles with white foreground), microenvironmental temperatures: substrate (C°, *T*_s_, white diamond) and air (°C; T_a_, white squares) and operative temperatures (°C; T_e_ grey triangles with black foreground) along the day (hour) of *Liolaemus montanezi.* Polygons connect the mean ± s.e.m. Wind velocity is indicated in the Y-right axis (m*s^−1^, white diamond with red foreground—whitish dashed line polygon) and percentages of relative humidity are indicated in the doble Y-right axis (RH %, black circle—black dashed line polygon).

T_b_s of *L. montanezi* were lower than T_s_, while T_b_ and T_s_ were significantly higher than T_a_ (Supplementary Table S2). T_b_s increased with T_a_s, and did not depend on T_s_s (Stepwise Regression, F_Tb-Ta (18)_ = 11.98, P < 0.001; F _Tb-Ts (18)_ = 8.95, P > 0.3, Fig. 1).

T_b_s were not significantly related to wind velocity, but it was negatively related to RH% (Simple Regression, F_Tb-Wd (1; 21)_ = 0.393, t = 0.627, R^2^ = 0.02, P > 0.5; F_Tb-RH% (1; 21)_ = 55.37, t =  − 7.442, R^2^ = 0.74, P  < 0.001, Fig. 1).

### Operative temperatures and thermal quality of the habitat

**Figure 2:**
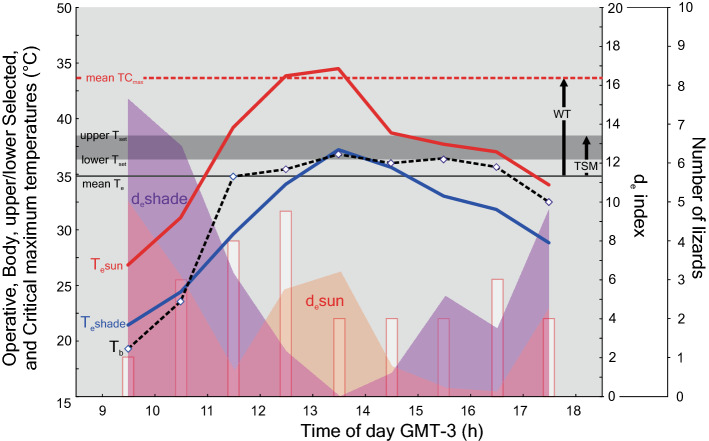
Variation during the activity of the mean operative temperatures obtained from open (°C, T_e_-sun: red solid line) and shaded (°C, T_e_-shade: blue solid line) habitats, and the mean body temperature (°C, T_b_: black dashed line with white diamonds—blue foreground) of *Liolaemus montanezi*. Mean operative temperatures (°C, T_e_: grey straight line), and critical thermal maximum (°C, CT_max_: dashed straight red line). The grey vanished area represents the set-point range of the preferred body temperature in laboratory (°C, upper and lower T_set_). The warming tolerance (WT) and the thermal safety margin for physiological performance (TSM) are indicated with arrows. The variation of the thermal quality during the activity calculated from open habitat (d_e_-sun index: faded red area) and shaded (d_e_-shade index: faded purple area) habitats are indicated in the Y-right axis. Number of lizards (faded white bars) are also indicated in the double Y-right axis.

Overall, the T_e_s were different between models exposed to sun or shad conditions (Two-way ANOVA, T_e_-sun vs. T_e_-shade, F_(1)_ = 786.4, P < 0.001), with the T_e_-sun higher than T_e_-shade (Supplementary Table S3, Fig. 2). Moreover, the hourly mean T_e_-sun was higher than the mean T_e_-shade (Two-way ANOVA, T_e_-sun vs. T_e_-shade * Hour F_(8)_ = 12.5, P < 0.001, Supplementary Table S3, Fig. 2).

Only 10% (N = 2) of the T_e_s records were included within T_set_, whereas 65% of T_e_s (N = 13) were lower than T_set_, and 25% (N = 5) of T_e_ exceeded the T_set_. However, the 38% (N = 8) of the operative temperatures in open habitat (T_e_-sun) exceeded the T_set_ from 11:00 to 15:00 h, whereas the operative temperatures from shaded habitat (T_e_-shade) were lower than the T_set_ of the species. Also, T_e_ exceeded CT_max_ during two hours in sunny habitats (Fig. 2).

Thermal quality (d_e_) was highly variable throughout the *L. montanezi* activity period, showing a trimodal distribution in open habitat (d_e_-sun), and a bimodal pattern in shaded habitats (d_e_-shade; Fig. 2). The mean thermal quality (d_e_) of the environment was lower in the sun than in the shade condition (d_e_-sun = 3.29 vs. d_e_-shade = 6.20).

### Preferred body temperatures, accuracy and effectiveness of thermoregulation

The mean preferred temperature for *L. montanezi* was 37.38 ± 1.53, whereas the lower and upper T_set_ obtained were 36.57 ± 1.94 °C and 38.40 ± 1.09 °C, respectively (N = 20; Fig. 2). A majority (65%, N = 13) of the T_b_s of *L. montanezi* were lower than the minimum T_set_, whereas 10% (N = 2) of the T_b_s were higher than the maximum T_set_. The remaining 25% of the T_b_’ records (N = 5) were within the T_set_ range. Males, females and juveniles of this species exhibit negative values of E, indicating there are other constraints that prevent them from thermoregulating within their set-points of T_pref_ (Table 1).

**Table 1:** Body size, thermal biology and thermal tolerance-related variables: Mean ± s.d. of body mass (BM, g), snout-vent length (SVL, mm), scaled mass index of body condition (M_i_), body temperatures (T_b_, °C), preferred body temperatures (T_pref_, °C), absolute values obtained from the individual deviation of T_b_ from T_set_ (d_b_), index of the average thermal quality of habitats (d_e_), and index of effectiveness of thermoregulation (E), voluntary thermal maximum (VT_max_), critical thermal maximum (CT_max_) and critical thermal minimum (CT_min_) between sexes and adult and juveniles of *L. montanezi*. The sample sizes N and (ranges) are also indicated.

Variables	Class			
	Males N = 9	Females N = 7	Juveniles N = 5	Overall N = 21
**Body size**				
BM	8.00 ± 0.84 (6.7 ‒ 9.5)	5.85 ± 0.47 (5.0 ‒ 6.4)	3.65 ± 1.45 (2.2 ‒ 5.27)	6.16 ± 1.97 (2.2 ‒ 9.5)
SVL	63.8 ± 2.70 (58.0 ‒ 67.5)	57.88 ± 1.02 (56.2 ‒ 59.0)	48.4 ± 5.50 (43.0 ‒ 54.0)	58.16 ± 6.88 (43.0 ‒ 67.5)
M_i_	5.83 ± 0.35 (5.26 ‒ 6.45)	5.95 ± 0.62 (4.8 ‒ 6.7)	6.09 ± 0.57 (5.39 ‒ 6.59)	5.94 ± 0.50 (4.8 ‒ 6.7)
**Thermal biology**				
T_b_	31.98 ± 4.85 (20.0 ‒ 35.6)	34.17 ± 6.62 (21.4 ‒ 40.0)	32.62 ± 7.82 (19.3 ‒ 38.0)	32.86 ± 5.98 (19.3 ‒ 40.0)
T_pref_	36.97 ± 1.59 (34.3 ‒ 38.8)	37.61 ‒ 1.99 (33.6 ‒ 39.4)	37.72 ± 0.50 (37.3 ‒ 38.5)	37.38 ± 1.53 (33.6 ‒ 39.4)
d_b_	4.60	4.43	4.86	4.60
d_e_	3.41	3.08	4.46	3.56
E	−0.34	−0.43	−0.08	−0.29
**Thermal tolerance**				
VT_max_	38.43 ± 2.95 (32.5 ‒ 41.8)	39.14 ± 2.57 (34.3 ‒ 41.8)	39.67 ± 1.72 (36.7 ‒ 41.1)	38.99 ± 2.49 (32.5 ‒ 41.8)
CT_max_	42.71 ± 1.61 (41.3 ‒ 46.1)	43.64 ± 1.12 (41.8 ‒ 45.1)	44.34 ‒ 1.17 (42.9 ‒ 46.2)	43.44 ± 1.44 (41.3 ‒ 43.4)
CT_min_	12.16 ± 1.18 (10.8 ‒ 14.0)	11.12 ± 1.16 (9.4 ‒ 12.8)	12.01 ‒ 0.44 (11.6 ‒ 12.7)	11.76 ± 1.10 (9.4 ‒ 14.0)

### Pairwise comparisons between T_b_ and T_pref_, and comparisons between T_b_, T_pref_, CT_max_, and CT_min_ among males, females and juveniles

The T_b_ of *L. montanezi* was significantly lower than T_pref_ (Wilcoxon, Paired rank test, T_(1; 20)_ = 34.000, z = 2.65; P > 0.006; Table 1). Thermophysiological variables, T_b_, T_pref_, CT_max_, and CT_min_ did not differ among males, females and juveniles (ANCOVA, T_b_, F_(2; 21)_ = 2.63, P < 0.1; T_pref_, F_(2; 20)_ = 0.97, P > 0.4; CT_max_ F_(2; 20)_ = 2.01, P > 0.17; CT_min_ F_(2; 20)_ = 2.32, P > 0.13; Table 1).

### Daily activity and microhabitat use: bare soil exposed to sun (BS-sun), bare soil in shade (BS-shade), and rocks in shade (WR-shade)

**Figure 3:**
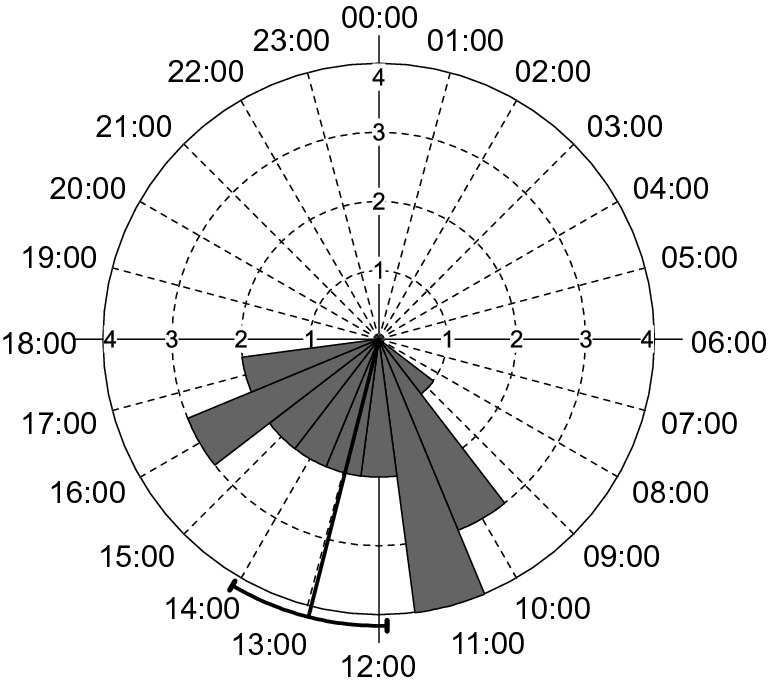
Circular histogram (rose diagram) representing the daily activity per 24-h of *Liolaemus montanezi*, corresponding to November 1, 2017. The bars represent the absolute frequencies of sighted lizards per hour. Vector and arc represent mean and the 95% confidence interval of the activity period.

*Liolaemus montanezi* (N = 21) was active for 9 h (09:00 to 18:00 h). Mean activity, [considered as the direction (vector) of the “Ɵ” angle and dispersion (± circular standard deviation) of the activity which is stretching within the origin to the end of the last observation] was at 12:58 h (Circular ± s.d. = 2 h 33 min = 38.261°, Fig. 3). Overall, the observed frequencies of individuals depicted a bimodal pattern of activity, with peaks occurring between 10:00 to 12:00 h in which 33% (N = 7) occurred and between 15:00 to 16:00 h, when 14% (N = 3) of lizards were seen during these time intervals (Fig. 3).

Nearly 62% (N = 13) of the observed lizards were recorded using BS-shade, 14% (N = 3) occupying BS-sun, and 24% (N = 5) associated to WR-shade microhabitats (Supplementary Table S4). Observed frequencies were different than expected among the microhabitats (Chi-square χ^2^ Test_2; 21_ = 8.0, P > 0.01), and the deviation of the expected frequencies indicated that BS-shade was more frequently used than BS-sun or WR-shade (Supplementary Fig. S1). However, when combined frequencies of temporal and spatial activity, the observed frequencies were marginally significantly higher than expected in the BS-sun at the 10:00 ‒ 11:00 h, and WR-shade at the 17:00 ‒ 18:00 h (Pearson Chi-Square χ^2^ Test _(16)_ = 29.47, P < 0.02; Multiple Comparison, z_BS-sun; 10-11 h_ = 2.66, P < 0.005; z _WR-shade;17-18 h_, = 9.08, P < 0.002, Supplementary Table S4), suggesting that lizards observed at these hours preferably occupies BS-sun and WR-shade microhabitats, respectively.

### Thermal tolerance, vulnerability to global warming, and extinction risk model

The mean critical thermal minimum, voluntary thermal maximum and critical thermal maximum were, CT_min_ = 11.76 ± 1.10, VT_max_ = 38.99 ± 2.49, and CT_max_ = 43.44 ± 1.44, respectively (Table 1). The indices to estimate the vulnerability to global warming were, WT = 8.8 °C and TSM = 3.57 °C (Fig. 2).

**Figure 4:**
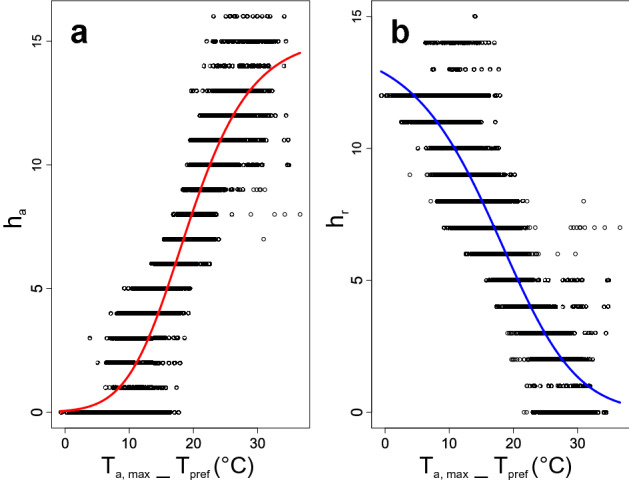
Richard´s Curve. Dependence of hours of activity (h_a_, panel a) and hours of restriction (h_r_, panel b) on daily maximum air temperature (T_a, max_) of *Liolaemus montanezi* along the year.

The h_a_ and h_r_ as a function of T_a, max_ of *L. montanezi* were explained by the logistic Richard’s curve (Table 2; Fig. 4). The parameters for the fitted functions of h_a_ and h_r_ for the present time and projected for the years 2050 and 2070 under RCP 4.5 and RPC 8.5 scenarios by months are shown in the Supplementary Table 5.

**Table 2:** Values of the parameters of the first half of Richard’s curve fitted for the hours of activity (h_a_) and hours of restriction (h_r_) on air maximum temperature (T_a, max_) of *L. montanezi*. A (asymptote), *k* (rate), *i* (inflection point) and *m* (shape) using nonlinear least squares (‘nls’ function R-stats) with ‘modpar’ and ‘SSposnegRichards’ of the R-package ‘FlexParamCurve’. The 95% c.i. were determined with the function ‘confint2’ of the R-package ‘nlstools’^108^. The *m* parameter is estimated from the data and fixed to allow others to behave as free parameters.

Parameters	h_a_, c.i. 95% (lower–upper)	h_r_, c.i. 95% (lower–upper)
A	15.136 (14.813–15.460)	13.955 (13.558–14.352)
*k*	0.169 (0.164–0.176)	− 0.134 (− 0.140 to − 0.128)
*i*	17.883 (17.646–18.120)	18.341 (17.929–18.752)
m*	0.410	0.549

**Figure 5:**
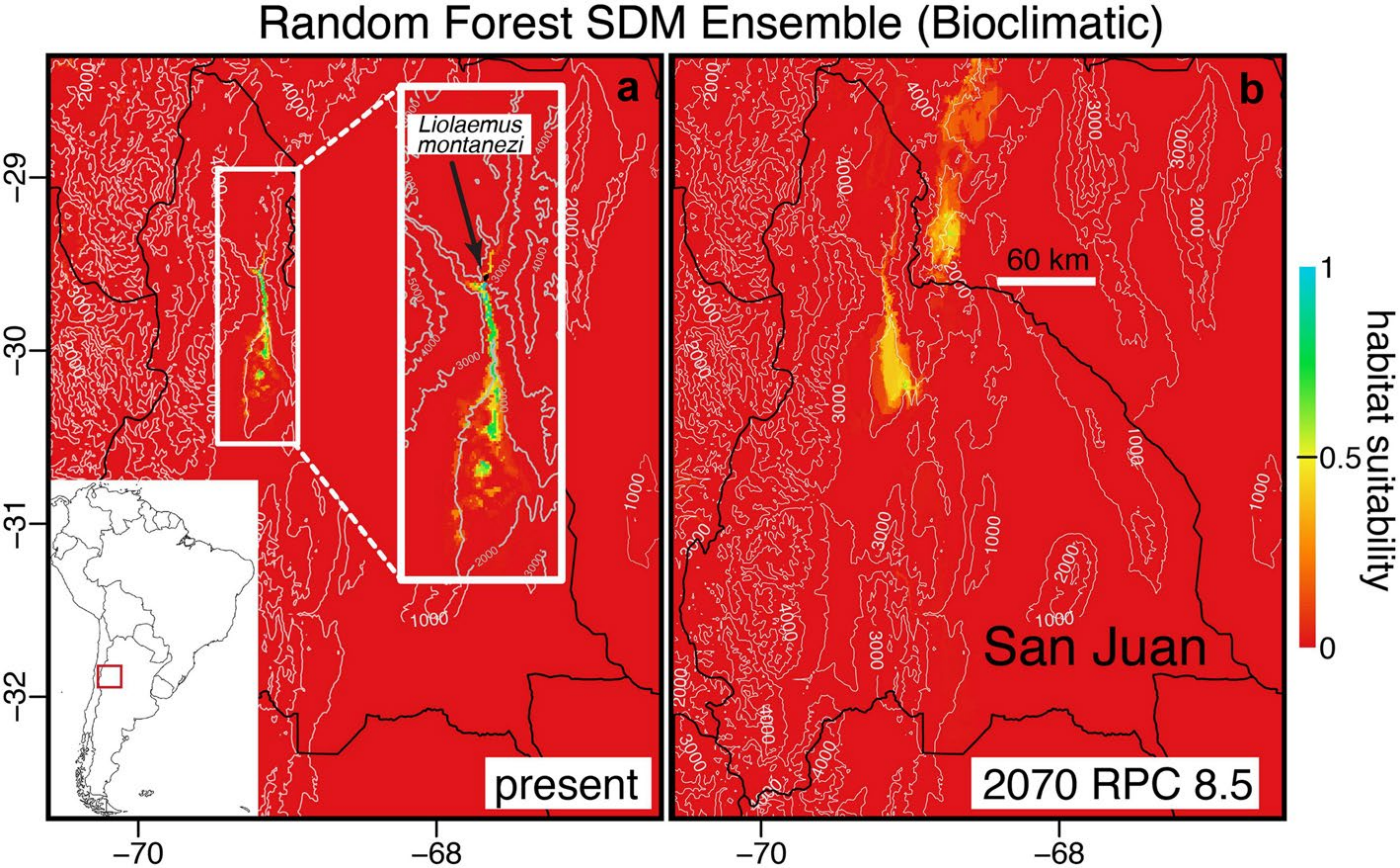
Suitable habitat projections for *L. montanezi* under bioclimatic layers and random forest algorithm (RF) at present (panel a) and 2070 RCP 8.5 (panel b). The inlay (white square) represents a close-up of the known distribution of the species. Elevation curves are also shown. These maps were generated in the R environment (R Core Team^109^ ver. 3.6.1, URL: http://www.r-project.org/index.html) using the R-packages: biomod2 ver. 3.4.6 (Thuiller et al.^110^, URL: https://CRAN.R-project.org/package=biomod2), dismo v1.1-4 (Hijmans et al.^111^, URL: https://CRAN.R-project.org/package=dismo), and raster v3.3-13 (Hijmans^112^, URL: https://CRAN.R-project.org/package=raster) by J.C. Santos.

**Figure 6:**
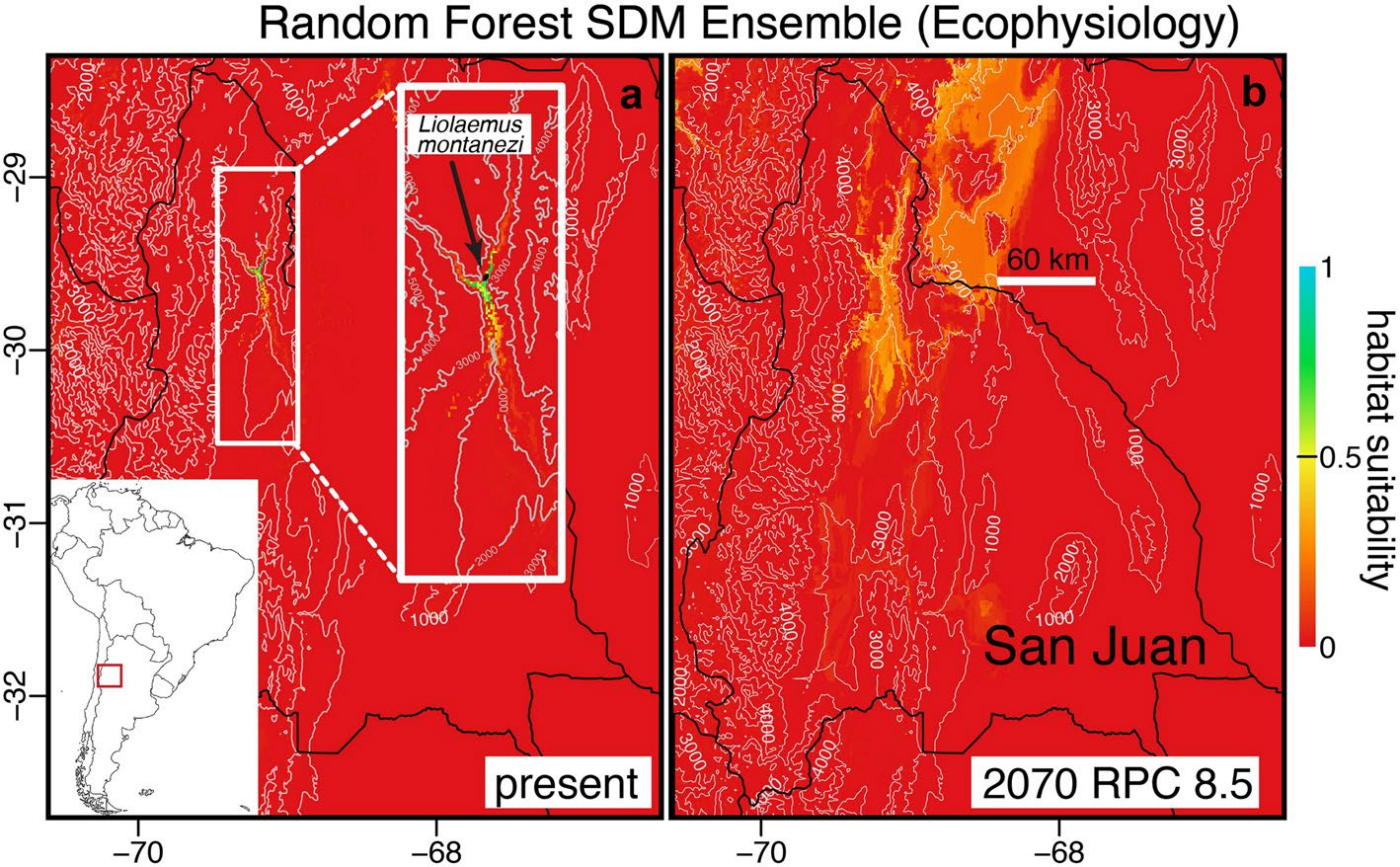
Suitable habitat projections for *L. montanezi* under ecophysiological layers and random forest algorithm (RF) at present (panel a) and 2070 RCP 8.5 (panel b). The inlay (white square) represents a close-up of the known distribution of the species. Elevation curves are also shown. These maps were generated in the R environment (R Core Team^109^ ver. 3.6.1, URL: http://www.r-project.org/index.html) using the R-packages: biomod2 ver. 3.4.6 (Thuiller et al.^110^, URL: https://CRAN.R-project.org/package=biomod2), dismo v1.1-4 (Hijmans et al.^111^, URL: https://CRAN.R-project.org/package=dismo), and raster v3.3-13 (Hijmans^112^, URL: https://CRAN.R-project.org/package=raster) by J.C. Santos.

Most evaluation statistics (ROC, TSS, KAPPA) showed that most models derived from each algorithm were very good (all > 0.75; Supplementary Table S7) to predict the current known distribution of *L. montanezi*. However, SDMs based on GAM failed for ecophysiological predictors, as well as some based on ANN (Supplementary Table S7). For projections, most SDMs indicated the locality where *L. montanezi* exists (Supplementary Figs. S2, S3). However, the ensemble derived from the RF algorithm was considered the most accurate model as it has fewer over-projections that describe the contemporary and future locations of habitat suitability for *L. montanezi* (Figs. 5, 6). Overall, the occupancy likelihood and habitat suitability modeled based on RF shows that the probable potential area with suitable climatic properties for *L. montanezi* represents a narrow stretch located toward the south of the actual distribution in the junction of Blanco and La Palca rivers (San Juan, Argentina) by 2050 and 2070, under 4.5 and 8.5 RCP (Figs. 5, 6).

## Discussion

Given the rapid decline and local extirpations of many lizards populations as consequence of climate change^13^, it has been emphasized that natural history traits and physiology are crucial to determine what critical environmental factors might be stressing and driving the decline in threatened species^63^. *Liolaemus montanezi* is a taxon that thermoregulates via a complex use of heat sources. Individuals were found basking directly exposed to the sun during the morning and retreat to shaded patches of *Bulnesia retama* and *Larrea divaricata* bushes to avoid overheating before midday during late spring. The mean T_s_ was significantly higher than T_b_ and T_a_ and, T_b_s only increased according to the air temperature, suggesting the usage of air temperature on thermoregulation^64,65^. Cooling by convection is common in some psammophilous species of *Liolaemus* inhabiting sandy environments from Brazil (*L. occipitalis*^66^; *L. lutzae*^67^; *L. arambarensis*^68^), and Argentina (*L. wiegmannii*^69^; *L. koslowskyi*^70^; *L. olongasta*^71^; *L. acostai*^72^; *L. sarmientoi*^73^; *L. ruibali*^74^; *L. chacoensis*^75^; *L. scapularis*^76^). Indeed, downslope winds with strong convection are common phenomena observed along the eastern slopes at medium or high elevations in the Andes, especially from midday and thenafter^77^. In the case of *L. montanezi*, the population is located in alluvial fans in the western margin and riverbed of the Blanco river, an open gully with steep slopes that results in a wind corridor. The convection of heat, rather than radiation or conduction, appears to be the main mechanism of heat loss that explains the T_b_ of *L. montanezi* (Fig. 1)*.*

In addition, body temperatures of *L. montanezi* varied according to the moisture level of the habitat. The inverse relationship between T_b_ and the percentage of relative humidity (RH %) suggests that the loss of environmental moisture at midday (12:00 h) might intensify the restriction of the activity, particularly in the sun-exposed microhabitats. Thus, when humidity decayed to near zero, lizards were frequently sighted using shaded habitats even when they still have high T_b_s in the shade (Figs. 1, 2). Sheltering under the bushes probably helps them to avoid dehydration which could also affect the accuracy of thermoregulation^78^. Furthermore, Nicholson et al.^79^ studying populations of *Anolis stratulus* and *Ameiva exsul* had pointed out that in water-restricted locations the interaction of temperature and humidity has strong influence on the daily activity patterns. Such environmental conditions have a noticeable demographic impact in many lizards since juveniles are more vulnerable to water loss than adults. Nevertheless, we found there were no differences in the ways in which males versus females, or juvenile versus adult lizards interact with their environment. The T_b_ of *L. montanezi* were not different between sexes and age classes, nor did T_b_ depend on body size or body condition. But, the CT_max_ was lower in lizards of larger body size, leading to the assumption of a more accurate thermoregulation in adults. Thus, we hypothesize that the drastic decline of humidity at 12:00 h might entice lizards to refuge in more suitable habitats that provide hiding shelters in shaded sites. During these restriction hours, lizards will wait until milder heat and humidity conditions are given to resume their daily active behavior.

These activity patterns usually reflect the extent in which the behavioral thermoregulation buffers the environmental restrictions to maintain the T_b_ to optimize physiological processes^80^. Thus, changes in ambient temperature during the day could cause the frequency of active lizards to describe a distribution that is unimodal or bimodal. Likewise, these patterns of behavior might also vary among the seasons of the year^80^. Hence, during spring *L. montanezi* might be active during 9 h from 09:00 to 18:00 h with activity peaks occurred between 10:00 to 12:00 h and between 15:00 to 16:00 h showing a bimodal pattern of activity (Figs. 2, 3) with a retreat during the time when the T_e_ max was higher than 44 °C (Fig. 2). When considering the operative temperatures of the models deployed over open habitats [i.e., those with high temperatures (T_e_-sun) or those placed in shaded habitats (T_e_-shade)], we found that they also support a pattern of activity likely associated with the thermal quality (d_e_ = d_e_-sun + d_e_-shade) of the environment (Fig. 2). Thus, we predict that the daily activity of these lizards starts when the environmental thermal quality is low (d_e_-sun and d_e_-shade exhibit the highest values) and increases while the thermal quality rises (d_e_-sun and d_e_-shade decrease). However, the number of active lizards outside shelters progressively decreases during the hotter hours and they are forced to retreat into shaded microhabitats where the thermal quality is optimal (d_e_-shade tending to zero). Based on our observations, we predict that a second peak of activity occurs in the afternoon when the thermal quality in shaded habitats decreases while it improves in open habitats allows the lizards to resume active behaviors (Fig. 2). Therefore, when considered as trimodal and bimodal patterns of d_e_-sun and d_e_-shade, respectively, both describe scenarios in which lizards emerge in the morning, retreat before the midday, and emerge again at the afternoon. This pattern of activity of *L. montanezi* during spring support the h_a_ and h_r_ observed considering the model for global warming (Fig. 4, Supplementary Table S5). Our operative temperature models likely provide more accurate representations of available temperatures at the capture than measurements of ambient air temperature, they do not represent "instantaneous" measures of equilibrium temperatures as recommended by Bakken et al^81^ and therefore our analyses involving operative temperatures should be interpreted as such.

Microhabitat selection by individuals may allow populations to maximize the geographic range^82^. In this sense, shrubs play a significant role in thermal ecology of the species. *Liolaemus montanezi* maintain the T_b_ near the operative temperatures recorded in the shaded habitats (Fig. 2), behaving as a thermoconformers during the hottest hours of the day. Thus, in a well-identified habitat, most of lizards were seen using sandy, bare soil in shaded places, sheltered beneath shrubs. However, when combining such microhabitat use with their diel activity pattern, we predicted that lizards would occupy strictly sunny, bare soil in the morning and would perch on rocks only in the afternoon (Supplementary Table S4). The fact that the individuals might use sunny bare soil at the beginning of the activity period, shuttling to cooler microhabitats during the warmest hours suggests that the species could exhibit spatial and temporal flexibility in microhabitat use^83^ but their thermoconformity in the shady refuges point out their current vulnerability.

Preferred temperature of *L. montanezi* during spring was higher than T_b_ as shown for other *Liolaemus* species^84^, even when the mean T_e_ was closer than T_set_ (Fig. 2). These results suggest that the species could obtain temperatures close to T_pref_ in their natural environment with low costs of thermoregulation. However, the effectiveness of thermoregulation (E =  − 0.29) shows that *L. montanezi* behaves almost as a thermoconformer (E = 0). But, the negative value shows that this species is avoiding thermally optimal microenvironments probably to minimize the risk of overheating^85^ and dehydration (during the warmest hours of the day) selecting among the coolest and shaded microhabitats. At least a third (33%) of the recorded T_e_ values were higher than the T_pref_ during the warmest hours which increases the risk for these lizards of attaining near-lethal body temperatures close to their CT_max_. Accordingly, we found that their thermal safety margin (TSM = 3.57 °C) and warming tolerance index (WT = 8.8) were notably lower than reported in others liolaemid lizards from cold temperate environments such as *Phymaturus tenebrosus* (TSM = 15.58 and WT = 23.3^86^) and *P. calcogaster* (TSM = 3.98 °C and WT = 10.67 °C^87^). Therefore, these observations suggest that *L. montanezi* has a narrow TSM range to prevent it from reaching lethal temperatures^45^. However, the thermal environments in the sub-Andean region characterized by high thermal variation could have a dramatic influence on such thermal tolerance. Thus, the mean breadth of thermal tolerance (CT_max_ − CT_min_, *sensu* Litmer & Murray^88^) for *L. montanezi* was 31.68 °C showing that this species is likely eurythermic.

The east side of the Andes, inhabited by *L. montanezi,* is expected to experience thermal alteration during global warming. An increase of T_a_ and a reduction of rainfall are expected to impose droughts and habitat modification^61,89^. Added thermal stress in the habitat is expected to cause a decline in the water availability and reduce plant growth and recruitment^61,90^. Hence, lizards, like *L. montanezi*, that typically use shady microhabitats to thermoregulate might experience temperature-driven activity restrictions^13^, which might compromise population persistence and cause local extirpations and threaten extinction^13,50^ in particular in microendemic taxa.

The current known distribution of *Liolaemus montanezi* is explained by abiotic and ecophysiological factors (Supplementary Table S6). As we pointed out above, the small area occupied by this species is confined between rivers to the southeast and steep slopes to the west and northwest. Because the known population of *L. montanezi* occupies sandy patches interspersed between alluvial fans in the western margin and riverbed of Blanco river, the availability of moisture as indicated by evapotranspiration (one of the predictors in the ecophysiological models) may play a main role in *L. montanezi* habitat physiognomy. The role of evapotranspiration is more conspicuous in the model than h_r_ and h_a_ for *L. montanezi* (Supplementary Table S6). Contemporary bioclimatic and ecophysiological models have produced comparable results. Hence, the SDM projections show a narrow range of suitable habitat for *L. montanezi* located along their current riverine habitats (La Palca and Blanco Rivers) southeast of their current range (Figs. 5, 6). Future SDMs predictions based on RF (Supplementary Table S7), includes the current range of *L. montanezi* with a higher probability of persistence by 2070 under RCP 8.5 (Figs. 5, 6).

The SDM reinforces the hypothesis of the river floodplain south of their current range serving as the more suitable habitat for the species in the future. However, numerous surveys were done in the predicted area of occupation and confirm that *L. montanezi* is not currently occupying these southern locations. The predicted pattern of habitat suitability is probably influenced by a fluvial effect of moisture supply which promotes the recruitment and persistence of the dominant plants used by *L. montanezi*. One alternative interpretation is that the model forecasts increased rainfall as projected between 2081–2100 by Barros et al.^61^ for that area. The MaxEnt, GAM, GLM, and ANN SDMs showed unreliable projections by overestimating the present and future scenarios localizing the species in currently unoccupied environments (Supplementary Figs. S2, S3). Instead, the RF approach projected a low likelihood of occupancy in neighboring areas in which the reduced range of this species will be intensified by adverse changes to its current habitats. Therefore, the microendemic character plus the strong dependence of this species on sandy patches covered with shrubs of *B. retama* and *L. divaricata,* together with the actual habitat fenced by the rivers suggest high constraints on niche tracking. This study also suggests the vulnerability of other species present in the area such as *L. eleodori*, *L. parvus*, and *Phymaturus punae.* The assessment of population vulnerability to climate change is a powerful tool to highlight how this and other species could become threatened within a relatively short time (~ 30 or 60 years). Our projections should be interpreted with caution given our low physiological sampling which were only held at the beginning of the activity season in spring. Despite of our modelled T_e_ obtained for 4123 days (see Supplementary material for details) the habitat suitability projections should be seen as preliminary. Additional work, with deeper sampling across more of the year, is necessary to fully understand the potential vulnerability to climate change in this species. This approach, although employing low sample size, provides enough species-specific ecophysiology and vulnerability information to prompt immediate reassessment of the conservation status of *L. montanezi*. Moreover, the SDM predictions can play an important role in support of a habitat conservation initiative. Although we cannot discard the notion of natural dispersal across the rivers to the south resulting in colonization of areas projected to be suitable in the future, prudent conservation would target new surveys of these areas to search for yet-undetected populations and to assess habitat quality for active translocation plans.

## Materials and methods

### Field work

#### Lizard sampling, study area, and climate

We captured a total of 21 specimens (9 males, 7 females, 5 juveniles) during one day in late spring (November) by noose in sandy environments in the riverbed at the NW margin of the junction of the Blanco and La Palca rivers, Iglesia Department, San Juan Province, Argentina (− 29.55 S; − 69.19 W, 2185 m asl). The study area belongs to the Monte phytogeographic region dominated by xerophilous plants such as shrubs of *Bulnesia retama*, *Larrea divaricata, Prosopis alpataco,* and *Atriplex crenatifolia*^91^. The climate corresponds to arid cold desert (BWk)^92^ with rainfall occurring mainly in the summer season and the annual mean temperature is < 18 °C.

#### Field data

Body temperatures (T_b_) were taken in active lizards using a catheter probe TP-K01 (1.62 mm diameter) introduced ca. 3 mm into the cloaca. Individuals were handled by the head to avoid heat transfer and the temperature was recorded within 20 s of handling. In order to evaluate the main heating resources used by *L. montanezi*, microenvironmental temperatures were recorded at the exact site of capture for each lizard: substratum temperatures sand, rocks or beneath dwarf shrubs (T_s,_ TP-K03 substrate probe), and air temperature at 1 cm above the ground (T_a_, TP-K02 gas probe). Probes were connected to a TES 1302 thermometer (TES, Electrical Electronic corp., Taipei, Taiwan, ± 0.01 °C). Thereafter, to determinate the relationship among environmental variables and lizard’s activity, we measured the wind velocity using an anemometer (Proster, ± 0.1 m/s) taken at 1 cm above the substrate at the exact first sighting of the lizard and we measured relative humidity (RH %; HOBO, Pro V2, HR%/T°C ± 2%). Snout-vent length (SVL, Mitutoyo, type Vernier digital caliper ± 0.01 mm) and body mass (BW, 10 g Pesola, spring scale ± 0.5 g) were also registered.

To determine the available spatiotemporal heterogeneity of the microenvironmental temperatures for thermoregulation, operative temperatures (T_e_, *sensu* Bakken^93^) were obtained using nine polyvinyl chloride (PVC) pipe models connected to external dataloggers (HOBO, U23-003, T°/T°C ± 1 °C) during the collection of lizards. The model with the greatest correspondence of thermal data to reflect the lizard T_b_ was a PVC pipe (80 mm length × 2.15 mm thickness) sealed at both ends with silicone Fastix (Regression: Adjusted R^2^ = 0.846, N = 2836, slope = 1.09, confidence interval = 1.05 ‒ 1.14)^94,95^. Subsequently, the models were deployed in the study area in the most representative microhabitats used by *L. montanezi*: (i) Bare soil with sunny, sandy substrate, (ii) Bare soil with shady, sandy substrate (beneath shrubs), and (iii) Weathered rocks in the shade beneath shrubs (3 microhabitats × 3 replicates). The T_e_ was recorded every 5 min during the lizard activity from 8:00 to 19:00 h during one day of fieldwork.

#### Activity pattern and microhabitat use

Daily activity and microhabitat use were recorded during one day throughout randomly visual surveys in ~ 2 km^2^ area. The survey was done walking from 08:00 to 19:00 h at a very slow pace (5‒6 m/min) to provide enough time to scan all available habitat. Then, we registered the number of active lizards (frequencies of lizards) and time of day of each lizard sighting. All lizards seen were captured to avoid any possibility of temporal pseudoreplication.

Due to the complexity of the habitat structure, the microhabitat used by the individuals were registered according to three main categories also used to deploy the T_e_ models, unifying all the suitable records as follows: (i) BS-sun or bare soil with sandy substrate exposed to sun when the lizards can be seen moving outside shelters; (ii) BS-shade or bare soil with a sandy substrate in the shade when the lizards can be seen perching in bushy-edges or sheltering beneath shrubs, and (iii) WR-shade or weathered rocks in the shade when the lizards were seen on shaded rocky substrate or on rocks in the shade.

### Laboratory experiments

We brought the lizards (N = 20; 8 males, 7 females, 5 juveniles) to the laboratory in individual cloth bags. Lizards were placed individually in open-top terraria with a sand substrate (120 cm length, 25 cm width, 30 cm height). Experiments were performed 2 days after captures. Because the photoperiod may affect the thermal physiology^96^, all experiments were performed within restricted periods corresponding to the range of activity of the species from 09:00 to 19:00 h. Lizards were supplied with water *ad libitum*, and fed with *Tenebrio* sp. larvae every day after the experiments.

#### Estimation of preferred body temperatures (T_pref_), and effectiveness of thermoregulation (E)

Lizards were provided with a thermal gradient produced by a 100-W incandescent lamp from 15° to 50 °C (taken 1 cm above the substrate) to thermoregulate during the experiments. Body temperatures were taken using ultra-thin (1 mm) catheter thermocouples located ca. 5 mm inside the cloaca and taped at the base of the lizard’s tail to prevent the thermocouple from being dislodged during the experiment. The temperature of each lizard was obtained every 5 min for 3 consecutive hours by connecting the thermocouple to an 8-channel data-logger (Measurement Computing 1.2 kHz Data Acquisition Device, OMEGA, TC-08 ± 0.5 °C, Stamford, CT, USA).

The preferred body temperatures registered for each individual during 3 h trial (T_pref-i_) were used to obtain the mean and the interquartile of T_pref-i_ for each individual (T_pref_ and T_set-i_, respectively). The accuracy of thermoregulation (d_b_) of *L. montanezi* in their natural environment, was calculated as the mean of the absolute values obtained from the deviations of T_b-i_ from T_set-i_ (individual deviation; d_b-i_). The index of the average thermal quality of the habitat from the organism’s perspective (d_e_) was calculated as the mean of the absolute values of the deviations of mean T_e_ (obtained within the hour of the capture for each lizard) from the T_set-i_. The effectiveness of temperature regulation, E, was calculated as 1 − (mean d_b-i_/mean d_e-i_)^38^. The values of E range from − 1 to 1, and E ~ 0 represents thermoconformers, E ~ 0.5 moderate thermoregulators, and E ~ 1 effective thermoregulators. Negative E values occur when lizards avoid thermally high-quality habitats with T_e_ near or within the range of T_pref_^85,97^. We also calculated the d_e_ in the shade (d_e_-shade) and d_e_ in the sun (d_e_-sun) in order to determine their differences in thermal quality, and to examine their contribution to the lizard’s activity.

#### Thermal tolerance: Determination of critical thermal minimum and maximum, and voluntary thermal maximum

Critical thermal minimum (CT_min_) and critical thermal maximum (CT_max_) were determined by means of cooling and heating trails, respectively. For CT_min_ and CT_max_, lizards were placed individually in a plastic transparent box (20 cm × 20 cm × 20 cm) with a layer (5 mm) of high-density Styrofoam covering the bottom of the box to prevent thermal conductance. Lizards were connected to ultra-thin K-type thermocouples (OMEGA, 5SC-TT-K-30-36; diameter = 0.076 mm, Norwalk, Connecticut, USA) introduced ca. ~ 5 mm inside the cloaca, and taped at the base of tail to prevent the thermocouple from being dislodged during the experiments, and T_b_ was recorded every 5 s using a data-logger (Measurement Computing 1.2 kHz Data Acquisition Device, OMEGA, TC-08 ± 0.5 °C, Stamford, CT, USA).

The lizards were placed individually in a 2 °C chest refrigerator with glass-top door cooled at constant rate (approximately, − 0.7 °C/30 s). During cooling, the lizards were turned onto their back (no more than four times per individual) until they reached CT_min_, considered as the T_b_ at which the lizard was no longer able to right itself when placed on its back^86^.

After at least 48 h, the heating experiments were performed. Lizards were heated at a constant rate (0.7 °C/10 s) using a 150-W infrared lamp placed 30 cm overhead. In the same experimental system, the voluntary thermal maximum (VT_max_) defined as the body temperature that induces a behavioral response seeking to cool down, was recorded^98^. Each lizard was monitored throughout the trials to record the critical thermal maximum (CT_max_), defined as the temperature at the higher extreme of tolerance in which the lizard was no longer able to right itself when placed onto its back^27,86,99^. Finally, after the determination of CT_min_, VT_max_ and CT_max_, each lizard was promptly removed from cooling or heating systems and steadily returned to room temperature to prevent the death of the individuals under study.

#### Vulnerability to global warming

To estimate population vulnerability to global warming we calculated the Warming Tolerance index (WT), defined as the difference between mean CT_max_ and mean T_e_ to show how close lizards are environmental temperatures to detrimental or lethal temperatures for physiological processes^86,100^. We also calculated the thermal safety margin (TSM) as the difference between mean T_pref max_ (upper T_pref_) and mean T_e_ (*sensu* Deutsch et al.^45^). Since most physiological processes are considered to occur at T_pref_, the upper T_pref_ threshold is a good proxy of the upper optimal temperature (T_opt_) for most physiological functions (i.e., digestion, locomotion or embryo development) that could exhibit a large degree of plasticity within the population^89^.

### Species distribution models (SDMs) using bioclimatic and ecophysiological predictors

We estimated the present and future habitat suitability derived from the known distribution locations for *L. montanezi* and its physiological parameters described above. For this purpose, we implemented two approaches for SDMs, first using generic bioclimatic method and secondly ecophysiological (species specific) predictors. The latter uses georeferenced grids (rasters) derived from predicted behavioral response of our focal species to environmental parameters and primary productivity estimates associated with the species’ habitats and community. We describe the SDM methods and their corresponding variable derivations in the Supplementary Material S1.

### Statistical analyses

Variability in thermophysiological variables was described using descriptive statistics (mean ± standard deviation, minimum and maximum). Normality and variance homogeneity assumptions were tested using Kolmogorov–Smirnov’s test and Levene’s test, respectively. When normality or variance homogeneity assumptions were not met, non-parametric correlation, Mann–Whitney, and Kruskal–Wallis rank sum were used^101^. Data were analyzed using Sigma Plot, version 14.0 (Systat Software Inc., San José, CA), SPSS, version 20.0 (IBM, SPSS Statistics for Windows, Armonk, NY), and figures were produced using Statistica, version 10.0 (Statsoft Inc., Tulsa, OK) and optimized with Corel X8, version 18.0 (Cowpland Research Lab, OTT, Canada).

Daily activity patterns were described using circular statistics. We estimate the mean vector (hour) and standard deviation among the frequencies of individuals with Oriana 4.02^102^. We also summarized all records plotting the frequencies of lizards sighted per hour using an angular histogram with Oriana 4.02^102^. The use of microhabitats was analyzed by contrasting the observed versus expected frequencies of lizards using a Chi-square test (χ^2^) under the assumption of uniformity in the expected frequencies (H_0_ = all microhabitat categories are equally used by lizards)^101^. Then, if the differences between observed versus expected frequencies were significant, a diagram of the deviation of the occurrence of individuals from its expected frequency (− 1 to 1 scale) was drawn to represent the relative changes of individuals among the microhabitats^103^.

A third approach that combined the periods of activity (by time of day) and microhabitat use (by categories of microhabitat) allowed the contrast of these two types of lizard frequencies. We then analyzed a 9 × 3 contingency table containing the frequencies of lizards by time intervals (N = 9, from 09:00 to 18:00 h) and microhabitat categories (N = 3, BS-sun, BS-shade, and WR-shade). Pearson's Chi-square test (χ^2^) was performed to evaluate significant differences between observed versus expected frequencies under assumption of uniformity in the expected frequencies (H_0_ = all microhabitat categories are equally used in any hour by the lizards). *Post hoc* analysis was performed from the adjusted residuals to obtain z-scores for each combined case. Subsequently, P-values were calculated from the transformation of Chi-square values derived from multiple tests^104,105^. Correction of Bonferroni was applied dividing the standard significance level (α = 0.05) by the number of multiple tests (*N* = 27, derived from 9 × 3 contingency table). Thus, the adjusted significance P-level obtained for the whole model was P < 0.0018.

Snout-vent length (SVL) and body mass (BW) were included in the scaled mass index of body condition in each individual (M_i_*, sensu* Peig and Green^106^) to determine the scaled mass index of condition as an indicator of the health or quality assumed to be related to fitness. The scale mass index was calculated as:

1 $$({\text{M}}_{\rm i} ) = {\text{M}}_{\rm i}^{*} [{\text{SVL}}_{\rm o} /{\text{SVL}}_{\rm i} ]\wedge {\text{bSMA}}$$

where M_i_ and SVL_i_ are the BW and SVL of each individual, SVL_0_ is the arithmetic mean SVL of the population, and bSMA is the standardized major axis slope from the regression of ln(BW) on ln(SVL) for the population^106^. The scaling bSMA exponent was calculated directly using the software RMA v. 1.21^107^. We then tested for the influence of M_i_ on thermal traits (T_b_, T_pref_, CT_max_, VT_max_, CT_min_) according to sex and age classes (males, females, and juveniles).

### Ethical statement

Capturing and handling of the lizards were conducted in accordance with international standards on animal welfare (ASIH/HL/SSAR Guidelines for Use of Live Amphibians and Reptiles), being compliant with Argentinian regulations (Argentinean National Law #14.346). All individuals were collected under permits Exp. Number 1300-2643. Field and laboratory protocols were approved in the UNSJ-SEADS-2017-RBSG research engagement and in the CICITCA-UNSJ 21/E1101 plan (Universidad Nacional de San Juan).

## Supplementary Information

Supplementary Information.

## Data Availability

All data needed to produce the results and discussion in the paper are present in the paper and/or the Supplementary Material.
