# Supplementary material for: Vulnerability to climate change of a microendemic lizard species from the central Andes
Source: Sci Rep. 2021 Jun 2;11:11653. doi: 10.1038/s41598-021-91058-w (PMC8172825; doi:10.1038/s41598-021-91058-w)
Supplement: Supplementary file 1 — Supplementary Information. [file 41598_2021_91058_MOESM1_ESM.docx]

**Vulnerability to climate change of a microendemic lizard species from the central Andes (Supplementary Information)**

Laspiur, A., Santos, J. C., Medina, S. M.; Pizarro, J. E.; Sanabria, E.A; Sinervo, B.; Ibargüengoytía, N. R.

**Supplementary Material S1.** Description of the Species Distribution models (SDMs) using bioclimatic and ecophysiological predictors

*Presences, absences and pseudoabsences*

The known distribution (i.e., true presences; N = 21) of *L. montanezi* was derived from all localities with registered individuals with museum vouchers and those localities with individuals observed and collected during our physiological and behavioral experiments. For each locality, we determined the coordinates (in degrees) and elevation (in m) using a Garmin, GPSmap 64s GPS receiver using the WGS84 datum. We also included museum records with geographic coordinates that have an associated vouchered type series specimen of *L. montanezi* ^1^. For true absences (N = 5), we visited several localities with the vicinity of known *L. montanezi* presences and if no individuals (or evidence of their presence) were determined and the geographic coordinates of such places were taken with the procedure as for true presences. For targeted absences (N = 47), we used records from localities in the vicinity where other *Liolaemus* species have been found, but these taxa are not sympatric and syntopic with to *L. montanezi*. This approach helps to minimize sampling bias in geographical (i.e., environmental) space and represent habitats that were competitive exclusion might have prevented *L. montanezi* to become local^2^. For pseudoabsences (N = 500), we used geographical points derived from a random selection with environmental profiling using true presences of *L. montanezi*. For this purpose, we use the function ‘OCSVMprofiling’ with present bioclimatic predictors as implemented in the R-package ‘mopa’ v 1.0.1^3^. This approach selects pseudoabsences with environmental profiling with one-classification support vector machine algorithms. The result is a selection of geographical points within the niche-space corresponding to areas of non-presence (outside the realized niche) of *L. montanezi*, but where movement factors are likely favorable to access such geographic areas. Finally, to minimize redundancy, we filtered (collapsed) points to a single geographic point within a proximity of 0.5 km radius (i.e., if two or more points are within this distance are collapsed to one) using ‘clean_points’ function of ‘Mapinguari’ R-package^4^ and we applied this procedure separately to all presences, absences and pseudoabsences.

*Bioclimatic and other climatological predictors*

We used the raster layers for the 19 bioclimatic variables at 30-second (~1 km) resolution of the WorldClim 2 dataset^5^. To estimate ecophysiological variables, we also obtained from the same repository the monthly average minimum and maximum temperatures (°C), and monthly total precipitation (mm). For all variables, we included future climatic projections for the CMIP5 data at 30-second spatial resolution for the MPI-ESM-LR for RCP (representative concentration pathways) 4.5 and 8.5 experiments predicted for years 2050 and 2070 also available WorldClim website (https://www.worldclim.org/). We recognize there are improve versions for WorldClim 2.1 (released on January, 2020), yet future projections have not been released for CMIP6 at 30-second resolution at the time of our modeling analyses.

*Ecophysiological predictors*

Long term historical collections of climate measurements at near soil levels does not exist for localities where *L. montanezi* has been collected. However, at least one long-term meteorological data collection exists from a nearby local weather station (San José de Jáchal, JCHL, ID-87305099999; 30.24259 S 68.74633 W) at 92 km south-eastern *L. montanezi* type locality that has similar climatological conditions. JCHL station has reported nearly continuously from 1973 to 2000 every-4-hour reports on air temperature, dew point, wind direction and speed, precipitation in METAR (Meteorological Aerodrome Reports) format. Such METAR daily reports were downloaded from the Surface Data Hourly Global (DS3505) archives (https://www.ncei.noaa.gov) for the JCHL station.

With the air temperature data for JCHL, we derived 24-hourly soil temperatures and the corresponding estimated hourly operative temperatures (T_e_, different than the T_e_ recorded at the capture using biophysical models during the fieldwork which were used to estimate d_e_ and E) for a lizard-shape ectotherm with snout-vent length of 58.17 mm (i.e., an average adult *L. montanezi* individual) at 0.5 cm above such soil. For this purpose, we used the following procedure. First, we discarded data for days that have <4-hour records through the day and < 40 records per month. This selection allowed us to remove days, months and years that could not be interpolated with enough confidence to obtain a 24-hour estimates air temperature for subsequent months. This data selection resulted in a total 4123 valid days. Second, with the retained data, we interpolated 24-hour periods using the function ‘interpolate_gaps_hourly’ from the R-package ‘chillR’ v 0.70.24^6^ with input of local latitude, available hour temperatures, and other default parameters for this function. Third, with the interpolated air temperature, we estimated the hourly temperature at the soil level (0.5 cm) using soil temperature functions (TsoilFunctions.R) with the parametrization of Beckman et al.^7^ implemented in the R-package ‘TrenchR’^8^. Finally, T_e_ was estimated for a lizard model implemented in the function (OperativeTemperatureFunctions.R) that uses the parametrization of Gates^9^ implemented also in the R-package ‘TrenchR’^8^.

With the estimated hourly T_e_, we determined the corresponding hours of activity (h_a_) and restriction (h_r_) for *L. montanezi* during its breeding (September-April) and non-breeding seasons. The determination if the animals will be active or restricted were determined based on field and lab observations with a lower limit for activity at T_b_ = 19.30°C and an upper limit at VT_max_ = 38.99°C (Table 1). Likewise, these lizards are diurnal and calculations where limited only to the light hours adjusted for latitude and the time of the year. This resulted in a binarized outcome where hourly activity where assigned to 1 (active) or 0 (inactive). At the end of each day, we summed the total (cumulative) hours of activity for the day (h_a_) and calculated its reciprocal (h_r_). With these parameters and the air temperature, we estimated the sigmoidal relationship between this temperature (x-axis) with h_a_ and h_r_ (y-axis) by fitting a positive-negative Richards (non-linear non-monotomic) curve with the function ‘modpar’ implemented in 'FlexParamCurve' v1.5-2^10^. This implementation of a Richards curve is as follows:

y = *A* / ([1 + *m* exp (- *k* (*x – i*))]^1/^*^m^*) + *A*’ / ([1 + *m’* exp (- *k’* (*x – i*))]^1/^*^m’^*), (1)

where y is h_r_ or h_a_ and x is T_a_ and four parameters are estimated simultaneously: *A* (asymptote), *k* (rate), *i* (inflection point) and *m* (shape). This parametrization is reduced to first half of the Richard´s curve equation (i.e., *A’*, *K’*, *I’* and *m’* are not estimated) which provides a standard equivalent to that in Sinervo et al.^11^. For details on the FlexParamCurve parametrization of the Richards curve see Oswald et al.^10^. We used the resulting Richards curve equation to estimate a raster grid using the maximum air temperature from WorldClim 2 dataset^5^. Finally, we estimated the monthly and annual potential and actual evapotranspiration (PET and AET) as implemented in ‘Mapinguari’ and ‘EcoHydRology’ R-packages^4,12^. The variables provide an approximate measure of hydric potential as the sum of evaporation from the land surface in addition to the transpiration from plants^13^.

*Estimating species distribution models (SDMs)*

Habitat suitability under current and future conditions were determined using the separated sets for bioclimatic and ecophysiological predictors. For this purpose, we used the filtered (collapsed) points for true presences, absences, and pseudoabsences. Before modeling, we subset each bioclimatic and ecophysiological to reduce multicollinearity using three approaches: (1) generalized boosted regression models with the ‘GBM’ R-package^14^; (2) regression with random forests with the ‘randomForest’ R-package^15^; and (3) variance inflation factor the ‘usdm’ R-package^16^. Only variables that contributed to the regressions and in consensus between these three methods where used for the subsequent analyses (Supplementary Table S6).

All SDMs for present and future projections were performed using ‘biomod2’ v 3.4.6 R-package^17^. For this purpose, we used the subset predictors and filtered (collapsed) points under a two-repetition scheme using GLM, GAM, ANN, RF, GBM and Maxent algorithms (see biomod2 vignette for details on each: http://search.r-project.org/library/biomod2/doc/Simple_species_modelling.pdf). We also split the point dataset into two independent subsets that include 75% of the points for training and 25% for testing then each of these two repetitions. A final model was derived for a global and each algorithm (e.g., GLMs, GBMs, RFs, etc) ensembles based on the weighted sum of probabilities of these two repetitions using their AUC metrics to determine their contribution to the global and per-algorithm ensembles (Supplementary Table S7). With these models, we estimated the projections using bioclimatic and ecophysiological predictors to the habitat suitability maps for *L. montanezi* under present and future RCPs 4.5 and 8.5 climatic conditions (Supplementary Figs. 2, 3). These projections were then plotted as maps to visualize the geographic areas of high and low suitability using the R-packages ‘raster’ and ‘dismo’^18,19^.

**Supplementary Table S1.** Results of linear regressions among body mass (BM), snout-vent length (SVL) and scaled mass index of body condition (M_i_) on body temperature (T_b_), preferred temperatures (T_pref_), voluntary thermal maximum (VT_max_), critical thermal maximum (CT_max_) and critical thermal minimum (CT_min_) of *Liolaemus montanezi*. Significant relationships are indicated with asterisk (*)

|  | Temperature | F | t | P |
| --- | --- | --- | --- | --- |
| BM | T_b_ | 1.230 | -1.109 | 0.2 |
|  | T_pref_ | 0.575 | -0.758 | 0.4 |
|  | VT_max_ | 0.07 | -0.277 | 0.7 |
|  | CT_max_ | 4.773 | -2.185 | 0.04* |
|  | CT_min_ | 0.004 | 0.06 | 0.9 |
| SVL | T_b_ | 0.980 | -0.990 | 0.3 |
|  | T_pref_ | 1.081 | -1.040 | 0.3 |
|  | VT_max_ | 0.738 | -0.859 | 0.4 |
|  | CT_max_ | 7.288 | -2.700 | 0.01* |
|  | CT_min_ | 0.229 | 0.478 | 0.6 |
| Mi | T_b_ | 0.602 | -0.776 | 0.4 |
|  | T_pref_ | 1.625 | 1.275 | 0.2 |
|  | VT_max_ | 4.876 | 2.208 | 0.04* |
|  | CT_max_ | 1.544 | 1.234 | 0.2 |
|  | CT_min_ | 4.200 | -2.049 | 0.05 |

**Supplementary Table S2.** Comparisons among body temperature (T_b_, °C), substrate (T_s_, °C) and air (T_a_, °C) temperature of *Liolaemus montanezi*. The mean ± SD, sample size (N), and the significance P < 0.05 with the symbol * are indicated. The One-way repeated measures ANOVA and the Holm-Šídák t-Test. Significant differences are indicated by asterisk (*).

|  | T_s_ | T_a_ |
| --- | --- | --- |
| T_b_ = 32.86 ± 5.98 (N = 21) | t = 4,817 * | t = 4,003 * |
| T_s_ = 36.75 ± 4.22 (N = 21) | ‒ | t = 8,820 * |
| T_a_ = 29.63 ± 3.64 (N = 21) | ‒ | ‒ |
| ANOVA, F_(df:2)_ = 39.006, P < 0.001 | | |

**Supplementary Table S3.** Summary of two-ways ANOVA analysis testing the differences between operative temperatures (*T_e_) between open (T_e_-sun) and shaded (T_e_-shade) habitats, and the differences between T_e_-sun and T_e_-shade within hours of activity from 9:00 to 18:00 h (Hour). Asterisks indicate statistical significance (P < 0.05). Mean ± s.d. and Range between parenthesis are also indicated.

| **Operative temperatures (T_e_)** | | | **Two-way**  **ANOVA** | |
| --- | --- | --- | --- | --- |
|  | **T_e_-sun** | **T_e_-shade** |  | |
| T_e_-sun vs. T_e_-shade | 37.7 ± 5.14  (25.5 ‒ 45.3) | 31.3 ± 4.53  (20.8 ‒ 38.7) | F _(1)_ = 786.4  P < 0.001* | |
| **Hour interval** | | | | |
| T_e_-sun vs. T_e_-shade  within the Hour | | | F _(8)_ = 12.5  P < 0.001* | |
| 9 ‒ 10 | 26,8 ± 0.88  (25.5 ‒ 27.7) | 21.4 ± 0.46  (20.8 ‒ 21.9) | t = 5.54 | P < 0.001* |
| 10 ‒ 11 | 31,1 ± 1.99  (28.2 ‒ 33.7) | 24.3 ± 1.44  (22.3 ‒ 26.4) | t = 10.66 | P < 0.001* |
| 11 ‒ 12 | 39.2 ± 2.94  (35.4 ‒ 44.5) | 29.6 ‒ 1.58  (27.1 ‒ 32.1) | t = 15.26 | P < 0.001* |
| 12 ‒ 13 | 43.8 ± 0.81  (42.2 ‒ 44.8) | 34.1 ± 1.17  (32.8 ‒ 36.1) | t = 15.51 | P < 0.001* |
| 13 ‒ 14 | 44.5 ± 0.53  (43.6 ‒ 45.3) | 37.2 ± 0.95  (35.7 ‒ 38.5) | t = 11.60 | P < 0.001* |
| 14 ‒ 15 | 38.7 ± 2.34  (37.2 ‒ 44.5) | 35.6 ±1.47  (34.6 ‒ 38.7) | t = 4.92 | P < 0.001* |
| 15 ‒ 16 | 37.6 ± 2.44  (32.9 ‒ 40.2) | 33.0 ± 0.57  (31.7 ‒ 34.1) | t = 7.40 | P < 0.001* |
| 16 ‒ 17 | 37.0 ± 1.04  (35.7 ‒ 39.0) | 31.7 ± 0.58  (30.8 ‒ 33.0) | t = 8.25 | P < 0.001* |
| 17 ‒ 18 | 34.0 ± 1.64  (31.6 ‒ 36.4) | 28.8 ± 0.83  (27.9 ‒ 30.1) | t = 8.22 | P < 0.001* |

**Supplementary Table S4.** Summary of Pearson’s Chi-Square χ^2^ test. Frequencies of lizards using the different microhabitat by hour. Microhabitat categories are BS-sun (bare sandy soil exposed to sun), BS-shade (bare sandy soil at shade), and WR-shade (weathered rocks at shade). *Post hoc* analysis are indicated by their adjusted z-scores (Adj. z-score) and probabilities (P). Bonferroni’s correction (*P* > 0.0018), and total sample sizes, and percentages are also indicated. Significant differences are in bold (*). Letters underlying P-values indicates equal or different expected frequencies of lizards among microhabitats by hour. Total frequencies and percentages of each categories are also indicated.

| Timetables of activity categories in hour (h) | | Pearson Chi-Square test, χ^2^ _(16)_ = 29.47, P < 0.02 | | | |
| --- | --- | --- | --- | --- | --- |
|  |  | Sun-BS | Shade-BS | Shade-WR | Frequency |
| 09:00 ‒ 10:00 | Frequency | 1 | 0 | 0 | 1 |
|  | Adj. z-score | 2.51 | -1.31 | -0.57 |  |
|  | P < 0.0018 | 0.01a | 0.19a | 0.56a |  |
| 10:00 ‒ 11:00 | Frequency | 2 | 1 | 0 | 3 |
|  | Adj. z-score | 2.66 | -1.10 | -1.05 |  |
|  | P < 0.0018 | **0.005b** | 0.27a | 0.29a |  |
| 11:00 ‒ 12:00 | Frequency | 0 | 4 | 0 | 4 |
|  | Adj. z-score | -0.91 | 1.74 | -1.24 |  |
|  | P < 0.0018 | 0.36a | 0.08a | 0.21a |  |
| 12:00 ‒ 13:00 | Frequency | 0 | 2 | 0 | 2 |
|  | Adj. z-score | -0.61 | 1.17 | -0.83 |  |
|  | P < 0.0018 | 0.54a | 0.24a | 0.40a |  |
| 13:00 ‒ 14:00 | Frequency | 0 | 1 | 1 | 2 |
|  | Adj. z-score | -0.61 | -0.36 | 0.91 |  |
|  | P < 0.0018 | 0.54a | 0.71a | 0.36a |  |
| 14:00 ‒ 15:00 | Frequency | 0 | 2 | 0 | 2 |
|  | Adj. z-score | -0.61 | 1.17 | -0.83 |  |
|  | P < 0.0018 | 0.54a | 0.24a | 0.40a |  |
| 15:00 ‒ 16:00 | Frequency | 0 | 2 | 0 | 2 |
|  | Adj. z-score | -0.61 | 1.17 | -0.83 |  |
|  | P < 0.0018 | 0.54a | 0.24a | 0.40a |  |
| 16:00 ‒ 17:00 | Frequency | 0 | 1 | 2 | 3 |
|  | Adj. z-score | -0.76 | -1.10 | 1.88 |  |
|  | P < 0.0018 | 0.44a | 0.27a | 0.06a |  |
| 17:00 ‒ 18:00 | Frequency | 0 | 0 | 2 | 2 |
|  | Adj. z-score | -0.61 | -1.9 | 9.08 |  |
|  | P < 0.0018 | 0.54a | 0.05a | **0.002b** |  |
| Total | Frequency | 3 | 13 | 5 | 21 |
|  | % | 14.3% | 61.9% | 23.8% | 100.0% |

**Supplementary Table S5.** Mean of numbers of hours of activity (h_a_) and hours of restrictions (h_r_) projected at the present, 2050 and 2070 under 4.5 and 8.5 RCP by months. Lower and upper 95% confidence interval (95% lower-upper) are indicated between parenthesis.

| Month | Present | | 2050 RCP 4.5 | | 2050 RCP 8.5 | | 2070 RCP 4.5 | | 2070 RCP 8.5 | |
| --- | --- | --- | --- | --- | --- | --- | --- | --- | --- | --- |
|  | h_a_ | h_r_ | h_a_ | h_r_ | h_a_ | h_r_ | h_a_ | h_r_ | h_a_ | h_r_ |
| Jan | 9.1  (9.0‒9.3) | 4.6  (4.5‒4.8) | 11.5  (11.4‒11.6) | 2.9  (2.7‒2.9) | 11.7  (11.6‒11.8) | 2.8  (2.6‒2.8) | 11.6  (11.5‒11.7) | 2.8  (2.6‒2.8) | 12.3  (12.2‒12.4) | 2.2  (2.1‒2.3) |
| Feb | 8.6  (8.5‒8.8) | 5.0  (4.9‒5.1) | 11.2  (11.1‒11.3) | 3.1  (3.0‒3.2) | 11.4  (11.3‒11.5) | 2.9  (2.8‒3.0) | 11.2  (11.1‒11.3) | 3.1  (3.0‒3.2) | 12.1  (11.9‒12.1) | 2.4  (2.3‒2.5) |
| Mar | 7.2  (7.0‒7.4) | 6.0  (5.9‒6.1) | 10.2  (10.1‒10.3) | 3.8  (3.7‒3.9) | 10.4  (10.3‒10.5) | 3.7  (3.6‒3.8) | 10.4  (10.3‒10.5) | 3.7  (3.6‒3.8) | 11.1  (11.0‒11.2) | 3.2  (3.1‒3.3) |
| Apr | 4.8  (4.7‒5.0) | 7.6  (7.5‒7.8) | 7.4  (7.3‒7.5) | 5.9  (5.8‒6.0) | 7.9  (7.8‒8.0) | 5.5  (5.4‒5.6) | 7.6  (7.5‒7.7) | 5.8  (5.7‒5.9) | 9.0  (8.9‒9.1) | 4.8  (4.7‒4.9) |
| Jun | 3.3  (3.2‒3.4) | 8.8  (8.7‒8.8) | 5.3  (5.2‒5.4) | 7.3  (7.2‒7.4) | 5.7  (5.6‒5.8) | 7.1  (7.0‒7.2) | 5.7  (5.6‒5.8) | 7.1  (7.0‒7.2) | 6.9  (6.8‒7.0) | 6.2  (6.1‒6.3) |
| Jul | 2.2  (2.2‒2.3) | 9.6  (9.5‒9.6) | 4.1  (4.0‒4.2) | 8.2  (8.1‒8.3) | 4.6  (4.5‒4.7) | 7.8  (7.7‒7.9) | 4.0  (3.9‒4.1) | 8.3  (8.2‒8.4) | 5.4  (5.3‒5.5) | 7.3  (7.2‒7.4) |
| Aug | 2.3  (2.2‒2.4) | 9.5  (9.5‒9.6) | 4.0  (3.9‒4.1) | 8.2  (8.1‒8.3) | 4.6  (4.5‒4.7) | 7.8  (7.7‒7.9) | 4.4  (4.3‒4.5) | 8.0  (7.9‒8.1) | 5.5  (5.4‒5.6) | 7.2  (7.1‒7.3) |
| Sep | 2.8  (2.7‒2.9) | 9.1  (9.0‒9.2) | 5.2  (5.1‒5.3) | 7.4  (7.3‒7.5) | 5.8  (5.7‒5.9) | 7.0  (6.9‒7.1) | 5.3  (5.2‒5.4) | 7.3  (7.2‒7.4) | 6.7  (6.6‒6.8) | 6.4  (6.3‒6.5) |
| Oct | 3.9  (3.7‒4.0) | 8.3  (8.2‒8.4) | 7.0  (6.9‒7.1) | 6.1  (6.0‒6.2) | 7.2  (7.1‒7.3) | 6.0  (5.9‒6.1) | 7.0  (6.9‒7.1) | 6.1  (6.0‒6.2) | 8.0  (7.9‒8.1) | 5.4  (5.3‒5.5) |
| Nov | 5.2  (5.0‒5.4) | 7.3  (7.2‒7.5) | 7.8  (7.7‒7.9) | 5.7  (5.5‒5.7) | 8.2  (8.1‒8.3) | 5.3  (5.2‒5.4) | 7.8  (7.7‒7.9) | 5.7  (5.5‒5.7) | 8.9  (8.8‒9.0) | 4.9  (4.7‒4.9) |
| Dec | 7.5  (7.3‒7.7) | 5.8  (5.7‒5.9) | 10.0  (9.9‒10.2) | 4.1  (3.9‒4.1) | 10.5  (10.3‒10.6) | 3.7  (3.5‒3.7) | 10.5  (10.3‒10.6) | 3.7  (3.5‒3.7) | 11.1  (11.0‒11.2) | 3.2  (3.0‒3.2) |

**Supplementary Table S6.** Results of multicollinearity analyses of bioclimatic and ecophysiological predictors using Generalized Boosted Machine (GBM), Random Forest (RF), Variance Inflation Factor (VIF) and Principal Components Analysis (PCA) selection methods. Asterisk (*) indicate significant relative influence for variables selection.

| **^a^ Bioclimatic variables** | | | | | |
| --- | --- | --- | --- | --- | --- |
| **BGM** | | **RF** | | **VIF** | |
| Var. | Rel.  Infl. | Var. | Rel.  Infl. | Var. | Rel. Infl. |
| BIO16 | 29.454* | BIO13 | 48.685* | BIO2 | 4.163* |
| BIO15 | 18.177* | BIO16 | 31.251* | BIO4 | 8.582* |
| BIO12 | 16.485* | BIO19 | 8.417* | BIO9 | 2.857* |
| BIO13 | 14.560* | BIO8 | 1.232* | BIO12 | 4.712* |
| BIO19 | 5.402* | BIO7 | 1.231* | BIO15 | 5.709* |
| BIO18 | 3.464* | BIO2 | 1.141* | BIO17 | 6.388* |
| BIO17 | 2.752 | BIO10 | 1.130 | BIO19 | 2.522* |
| BIO4 | 2.335 | BIO4 | 0.991 | BIO5 | -- |
| BIO11 | 1.391 | BIO5 | 0.936 | BIO10 | -- |
| BIO2 | 1.099 | BIO15 | 0.851 | BIO1 | -- |
| BIO8 | 0.909 | BIO3 | 0.767 | BIO7 | -- |
| BIO7 | 0.727 | BIO18 | 0.665 | BIO16 | -- |
| BIO6 | 0.631 | BIO14 | 0.492 | BIO11 | -- |
| BIO5 | 0.624 | BIO1 | 0.490 | BIO18 | -- |
| BIO14 | 0.589 | BIO12 | 0.472 | BIO3 | -- |
| BIO1 | 0.530 | BIO9 | 0.429 | BIO13 | -- |
| BIO9 | 0.519 | BIO17 | 0.319 | BIO6 | -- |
| BIO10 | 0.296 | BIO6 | 0.246 | BIO14 | -- |
| BIO3 | 0.046 | BIO11 | 0.246 | BIO8 | -- |
| **^b^Ecophysiological variables** | | | | | |
| **BGM** | | **RF** | | **VIF** | |
| Var. | Rel. Infl. | Var. | Rel. Infl. | Var. | Rel. Infl. |
| non_Breeding_AET | 63.389* | non_Breeding_AET | 6.15e+01* | Breeding_AET | 4.52* |
| non_Breeding_prec | 7.684* | non_Breeding_rh_raster_x_1000 | 6.23e+00* | non_Breeding_ha_raster_x_100 | 2.44* |
| rh_year_mean_x_10000 | 4.331* | Breeding_hr_raster_x_100 | 5.90e+00* | Breeding_rh_raster_x_1000 | 1.88* |
| Breeding_rh_raster_x_1000 | 4.330* | rh_year_mean_x_10000 | 5.71e+00* | non_Breeding_AET | 1.85* |
| total_AET | 4.097* | non_Breeding_prec | 3.25e+00* | rh_year_mean_x_10000 | 1.19* |
| Breeding_prec | 4.007 | Breeding_ha_raster_x_100 | 3.23e+00 | total_prec | -- |
| total_prec | 3.983 | Breeding_AET | 3.20e+00 | total_AET | -- |
| Breeding_AET | 2.011 | total_AET | 3.06e+00 | h_a_year_x_100 | -- |
| Breeding_hr_raster_x_100 | 1.774 | Breeding_prec | 2.89e+00 | h_r_year_x_100 | -- |
| non_Breeding_rh_raster_x_1000 | 1.772 | total_prec | 2.78e+00 | non_Breeding_rh_raster_x_1000 | -- |
| Breeding_ha_raster_x_100 | 1.181 | Breeding_rh_raster_x_1000 | 8.60e-01 | non_Breeding_hr_raster_x_100 | -- |
| non_Breeding_ha_raster_x_100 | 0.673 | non_Breeding_hr_raster_x_100 | 5.45e-01 | Breeding_ha_raster_x_100 | -- |
| non_Breeding_hr_raster_x_100 | 0.282 | h_r_year_x_100 | 5.13e-01 | Breeding_prec | -- |
| h_r_year_x_100 | 0.254 | h_a_year_x_100 | 2.58e-01 | Breeding_hr_raster_x_100 | -- |
| h_a_year_x_100 | 0.224 | non_Breeding_ha_raster_x_100 | 5.52e-15 | non_Breeding_prec | -- |

^a^BIO1 = Annual mean temperature; BIO2 = Mean diurnal range (Mean of monthly (max temp - min temp)); BIO3 = Isothermality (BIO2/BIO7) (×100); BIO4 = Temperature seasonality (standard deviation ×100); BIO5 = Max temperature of warmest month; BIO6 = Min temperature of coldest month; BIO7 = Temperature annual range (BIO5-BIO6); BIO8 = Mean Temperature of wettest quarter; BIO9 = Mean temperature of driest quarter; BIO10 = Mean temperature of warmest quarter; BIO11 = Mean temperature of coldest quarter; BIO12 = Annual precipitation; BIO13 = Precipitation of wettest month; BIO14 = Precipitation of driest month; BIO15 = Precipitation seasonality (Coefficient of Variation); BIO16 = Precipitation of wettest quarter; BIO17 = Precipitation of driest quarter; BIO18 = Precipitation of warmest quarter; BIO19 = Precipitation of coldest quarter.

^b^non_Breeding_AET = actual evaporation at non-breeding season; non_Breeding_prec: precipitation at non-breeding season; rh_year_mean_x_10000 = mean of annual relative humidity x 10000; Breeding_rh_raster_x_1000 = relative humidity at breeding season x 1000; total_AET = total actual evapotranspiration; Breeding_prec = precipitation at breeding season; total_prec = total precipitation; Breeding_AET = actual evapotranspiration at breeding season; Breeding_hr_raster_x_100 = hours of restriction at breeding season x 100; non_Breeding_rh_raster_x_1000 = relative humidity raster at non-breding season x 1000; Breeding_ha_raster_x_100 = hours of activity at breeding season x 100; non_Breeding_ha_raster_x_100 = hours of activity at non-breeding season; non_Breeding_hr_raster_x_100 = hours of restriction at non-breeding season; h_r_year_x_100 = annual hours of restriction x 100; h_a_year_x_100 = annual hours of activity x 100.

**Supplementary Table S7.** Assessment of accuracy of extinction risk models based on bioclimatic and ecophysiological data for *L. montanezi* using different algorithms (GLM, GAM, ANN, GBM, RF and MaxEnt).

| algorithm^a^ | Evaluation metric^b^ | Run evaluation | Bioclimatic model | | | | Ecophysiological model | | | |
| --- | --- | --- | --- | --- | --- | --- | --- | --- | --- | --- |
|  |  |  | Testing data | Cut-off | Sensitivity | Specificity | Testing data | Cut-off | Sensitivity | Specificity |
| GLM | ROC | RUN1 – PA1 | 0.995 | 500 | 100 | 98.958 | 1 | 500 | 100 | 100 |
|  |  | RUN2 ‒ PA1 | 0.875 | 500.5 | 75 | 100 | 0.988 | 500 | 100 | 97.569 |
|  |  | RUN1 ‒ PA2 | 0.873 | 500 | 75 | 99.653 | 0.981 | 500 | 100 | 96.181 |
|  |  | RUN2 ‒ PA2 | 1 | 500 | 100 | 100 | 1 | 550.5 | 100 | 100 |
|  | TSS | RUN1 – PA1 | 0.99 | 495 | 100 | 98.958 | 1 | 495 | 100 | 100 |
|  |  | RUN2 ‒ PA1 | 0.75 | 500 | 75 | 100 | 0.976 | 495 | 100 | 97.569 |
|  |  | RUN1 ‒ PA2 | 0.747 | 495 | 75 | 99.653 | 0.962 | 495 | 100 | 96.181 |
|  |  | RUN2 ‒ PA2 | 1 | 495 | 100 | 100 | 1 | 545 | 100 | 100 |
|  | KAPPA | RUN1 – PA1 | 0.722 | 495 | 100 | 98.958 | 1 | 495 | 100 | 100 |
|  |  | RUN2 ‒ PA1 | 0.855 | 500 | 75 | 100 | 0.524 | 495 | 100 | 97.569 |
|  |  | RUN1 ‒ PA2 | 0.747 | 495 | 75 | 99.653 | 0.408 | 495 | 100 | 96.181 |
|  |  | RUN2 ‒ PA2 | 1 | 495 | 100 | 100 | 1 | 545 | 100 | 100 |
| GAM | ROC | RUN1 – PA1 | 0.997 | 980 | 100 | 99.306 | NA | NA | NA | NA |
|  |  | RUN2 ‒ PA1 | 0.873 | 500 | 75 | 99.653 | 1 | 355 | 100 | 100 |
|  |  | RUN1 ‒ PA2 | 0.873 | 500 | 75 | 99.653 | 0.998 | 999 | 100 | 99.653 |
|  |  | RUN2 ‒ PA2 | NA | NA | NA | NA | NA | NA | NA | NA |
|  | TSS | RUN1 – PA1 | 0.993 | 975 | 100 | 99.306 | NA | NA | NA | NA |
|  |  | RUN2 ‒ PA1 | 0.747 | 495 | 75 | 99.653 | 1 | 354 | 100 | 100 |
|  |  | RUN1 ‒ PA2 | 0.747 | 495 | 75 | 99.653 | 0.993 | 975 | 100 | 99.306 |
|  |  | RUN2 ‒ PA2 | NA | NA | NA | NA | NA | NA | NA | NA |
|  | KAPPA | RUN1 – PA1 | 0.797 | 975 | 100 | 99.306 | NA | NA | NA | NA |
|  |  | RUN2 ‒ PA1 | 0.747 | 495 | 75 | 99.653 | 1 | 354 | 100 | 100 |
|  |  | RUN1 ‒ PA2 | 0.747 | 495 | 75 | 99.653 | 0.797 | 975 | 100 | 99.306 |
|  |  | RUN2 ‒ PA2 | NA | NA | NA | NA | NA | NA | NA | NA |
| ANN | ROC | RUN1 – PA1 | 1 | 994 | 100 | 100.000 | NA | NA | NA | NA |
|  |  | RUN2 ‒ PA1 | 0.993 | 57.5 | 100 | 97.569 | 0.997 | 64 | 100 | 99.306 |
|  |  | RUN1 ‒ PA2 | 0.911 | 998.5 | 75 | 100.000 | 0.998 | 499.5 | 100 | 99.653 |
|  |  | RUN2 ‒ PA2 | 1 | 500 | 100 | 100 | 1 | 500 | 100 | 100 |
|  | TSS | RUN1 – PA1 | 1 | 989 | 100 | 99.653 | NA | NA | NA | NA |
|  |  | RUN2 ‒ PA1 | 0.747 | 541 | 75 | 99.653 | 0.993 | 67 | 100 | 99.306 |
|  |  | RUN1 ‒ PA2 | 0.747 | 536 | 75 | 99.653 | 0.997 | 494 | 100 | 99.653 |
|  |  | RUN2 ‒ PA2 | 1 | 495 | 100 | 100 | 1 | 495 | 100 | 100 |
|  | KAPPA | RUN1 – PA1 | 1 | 989 | 100 | 99.653 | NA | NA | NA | NA |
|  |  | RUN2 ‒ PA1 | 0.747 | 541 | 75 | 99.653 | 0.797 | 67 | 100 | 99.306 |
|  |  | RUN1 ‒ PA2 | 0.747 | 536 | 75 | 99.653 | 0.887 | 494 | 100 | 99.653 |
|  |  | RUN2 ‒ PA2 | 1 | 495 | 100 | 100 | 1 | 495 | 100 | 100 |
| GBM | ROC | RUN1 – PA1 | 1 | 736 | 100 | 100 | 1 | 407 | 100 | 100 |
|  |  | RUN2 ‒ PA1 | 0.999 | 175.5 | 100 | 99.653 | 0.868 | 607 | 75 | 100 |
|  |  | RUN1 ‒ PA2 | 0.872 | 526 | 75 | 100 | 1 | 592.5 | 100 | 100 |
|  |  | RUN2 ‒ PA2 | 1 | 621.5 | 100 | 100 | 1 | 609 | 100 | 100 |
|  | TSS | RUN1 – PA1 | 1 | 736 | 100 | 100 | 1 | 406.5 | 100 | 100 |
|  |  | RUN2 ‒ PA1 | 0.997 | 178 | 100 | 99.653 | 0.75 | 608 | 75 | 100 |
|  |  | RUN1 ‒ PA2 | 0.75 | 526.5 | 75 | 100 | 1 | 592 | 100 | 100 |
|  |  | RUN2 ‒ PA2 | 1 | 624 | 100 | 100 | 1 | 606 | 100 | 100 |
|  | KAPPA | RUN1 – PA1 | 1 | 736 | 100 | 100 | 1 | 406.5 | 100 | 100 |
|  |  | RUN2 ‒ PA1 | 0.887 | 178 | 100 | 99.653 | 0.855 | 608 | 75 | 100 |
|  |  | RUN1 ‒ PA2 | 0.855 | 526.5 | 75 | 100 | 1 | 592 | 100 | 100 |
|  |  | RUN2 ‒ PA2 | 1 | 624 | 100 | 100 | 1 | 606 | 100 | 100 |

**Supplementary Table 7 Continued**

| RF | ROC | RUN1 – PA1 | 1 | 685.5 | 100 | 100 | 1 | 321 | 100 | 100 |
| --- | --- | --- | --- | --- | --- | --- | --- | --- | --- | --- |
|  |  | RUN2 ‒ PA1 | 0.999 | 194 | 100 | 99.653 | 0.989 | 7 | 100 | 95.139 |
|  |  | RUN1 ‒ PA2 | 0.999 | 37.5 | 100 | 99.653 | 1 | 628.5 | 100 | 100 |
|  |  | RUN2 ‒ PA2 | 1 | 532.5 | 100 | 100 | 1 | 514.5 | 100 | 100 |
|  | TSS | RUN1 – PA1 | 1 | 687 | 100 | 100 | 1 | 323 | 100 | 100 |
|  |  | RUN2 ‒ PA1 | 0.997 | 192 | 100 | 99.653 | 0.889 | 0 | 100 | 0 |
|  |  | RUN1 ‒ PA2 | 0.997 | 35 | 100 | 99.653 | 1 | 630 | 100 | 100 |
|  |  | RUN2 ‒ PA2 | 1 | 530 | 100 | 100 | 1 | 514 | 100 | 100 |
|  | KAPPA | RUN1 – PA1 | 1 | 687 | 100 | 100 | 1 | 323 | 100 | 100 |
|  |  | RUN2 ‒ PA1 | 0.887 | 192 | 100 | 99.653 | 0.855 | 500 | 75 | 100.000 |
|  |  | RUN1 ‒ PA2 | 0.887 | 35 | 100 | 99.653 | 1 | 630 | 100 | 100 |
|  |  | RUN2 ‒ PA2 | 1 | 530 | 100 | 100 | 1 | 514 | 100 | 100 |
| MaxEnt | ROC | RUN1 – PA1 | 0.998 | 327 | 100 | 99.653 | 0.988 | 250 | 100 | 97.569 |
|  |  | RUN2 ‒ PA1 | 0.998 | 308 | 100 | 99.653 | 1 | 288 | 100 | 100 |
|  |  | RUN1 ‒ PA2 | 1 | 460.5 | 100 | 100 | 1 | 341 | 100 | 100 |
|  |  | RUN2 ‒ PA2 | 0.998 | 250 | 100 | 99.653 | 1 | 250 | 100 | 100 |
|  | TSS | RUN1 – PA1 | 0.997 | 324 | 100 | 99.653 | 0.976 | 247 | 100 | 97.569 |
|  |  | RUN2 ‒ PA1 | 0.997 | 305 | 100 | 99.653 | 1 | 285 | 100 | 100 |
|  |  | RUN1 ‒ PA2 | 1 | 458 | 100 | 100 | 1 | 338 | 100 | 100 |
|  |  | RUN2 ‒ PA2 | 0.997 | 247 | 100 | 99.653 | 1 | 247 | 100 | 100 |
|  | KAPPA | RUN1 – PA1 | 0.887 | 324 | 100 | 99.653 | 0.524 | 247 | 100 | 97.569 |
|  |  | RUN2 ‒ PA1 | 0.887 | 305 | 100 | 99.653 | 1 | 285 | 100 | 100 |
|  |  | RUN1 ‒ PA2 | 1 | 458 | 100 | 100 | 1 | 338 | 100 | 100 |
|  |  | RUN2 ‒ PA2 | 0.887 | 247 | 100 | 99.653 | 1 | 247 | 100 | 100 |

^a^GLM = generalized linear model; GAM = Generalized Additive Model; ANN = Artificial Neural Network; GBM = Generalized Boosted Machine; RF = Random Forest; MaxEnt = Maximum entropy.

^b^ ROC = Receiver operating characteristic; TSS = true skill statistic; KAPPA = kappa statistic.

**Supplementary Fig. S1.** Diagram of deviation of expected frequencies under the hypothesis of uniformity of the use of microhabitat. Gray bars indicate the deviation of the expected frequencies transformed to -1 to 1 scale. BS-sun: bare sandy soil exposed to sun), BS-shade: bare sandy soil at shade, and WR-shade: weathered rocks at shade.


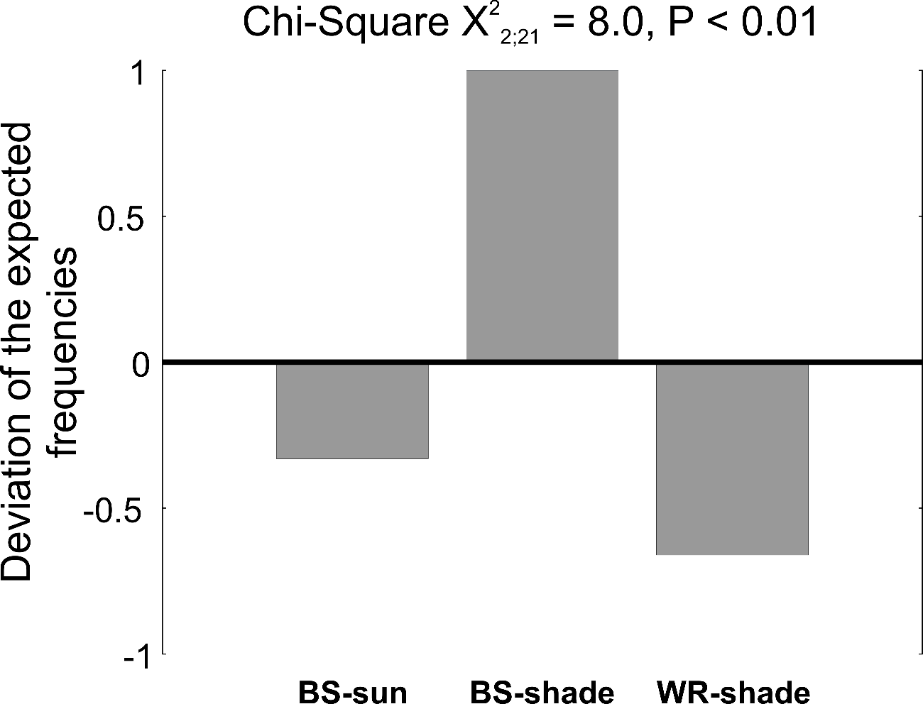


**Supplementary Fig. S2.** Occupancy likelihood of bioclimatic model for the present (1A to 1G), 2050 (RCP 4.5 = 2A to 2G and RCP 8.5 = 4A to 4G) and 2070 (RCP 4.5 = 3A to 3G, and RCP 8.5 = 5A to 5G) under RCP sceneries. Black circles represent all known *L. montanezi* localities. Warmer colors symbolize a low occupancy likelihood, or high extinction risk. (A) Summary plot of all algorithm together (A), Maximum entropy, MAXENT (B), Summary plot of all algorithm except MaxEnt (C), Artificial Neural Network, ANN (D), Generalized Additive Model, GAM (E), Generalized Boosted Machine, BGM (F), generalized linear model, GLM (G) and Random Forest, FR (H). These maps were generated in the R environment (R Core Team^20^ ver. 3.6.1, URL: http://www.r-project.org/index.html) using the R-packages: biomod2 ver. 3.4.6 (Thuiller et al.^17^, URL: https://CRAN.R-project.org/package=biomod2), dismo v1.1-4 (Hijmans et al.^19^, URL: https://CRAN.R-project.org/package=dismo), and raster v3.3-13 (Hijmans^18^, URL: https://CRAN.R-project.org/package=raster) by J.C. Santos.


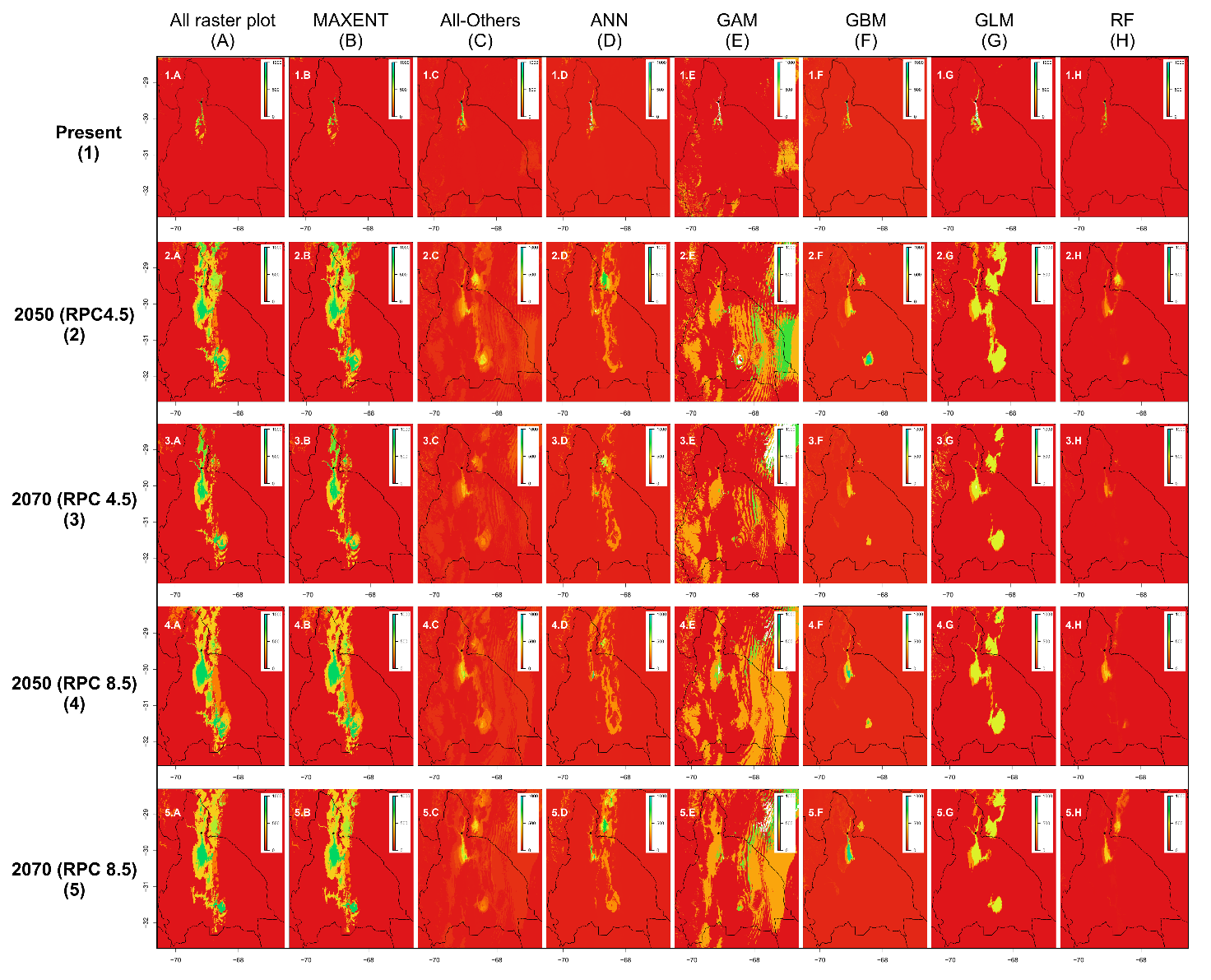


**Supplementary Fig. S3.** Occupancy likelihood of ecophysiological model for the present (1A to 1G), 2050 (RCP 4.5 = 2A to 2G and RCP 8.5 = 4A to 4G) and 2070 (RCP 4.5 = 3A to 3G, and RCP 8.5 = 5A to 5G) under RCP sceneries. Black circles represent all known *L. montanezi* localities. Warmer colors symbolize a low occupancy likelihood, or high extinction risk. (A) Summary plot of all algorithm together (A), Maximum entropy, MAXENT (B), Summary plot of all algorithm except MaxEnt (C), Artificial Neural Network, ANN (D), Generalized Boosted Machine, BGM (E), generalized linear model, GLM (F) and Random Forest, FR (G). These maps were generated in the R environment (R Core Team^20^ ver. 3.6.1, URL: http://www.r-project.org/index.html) using the R-packages: biomod2 ver. 3.4.6 (Thuiller et al.^17^, URL: https://CRAN.R-project.org/package=biomod2), dismo v1.1-4 (Hijmans et al.^19^, URL: https://CRAN.R-project.org/package=dismo), and raster v3.3-13 (Hijmans^18^, URL: https://CRAN.R-project.org/package=raster) by J.C. Santos.


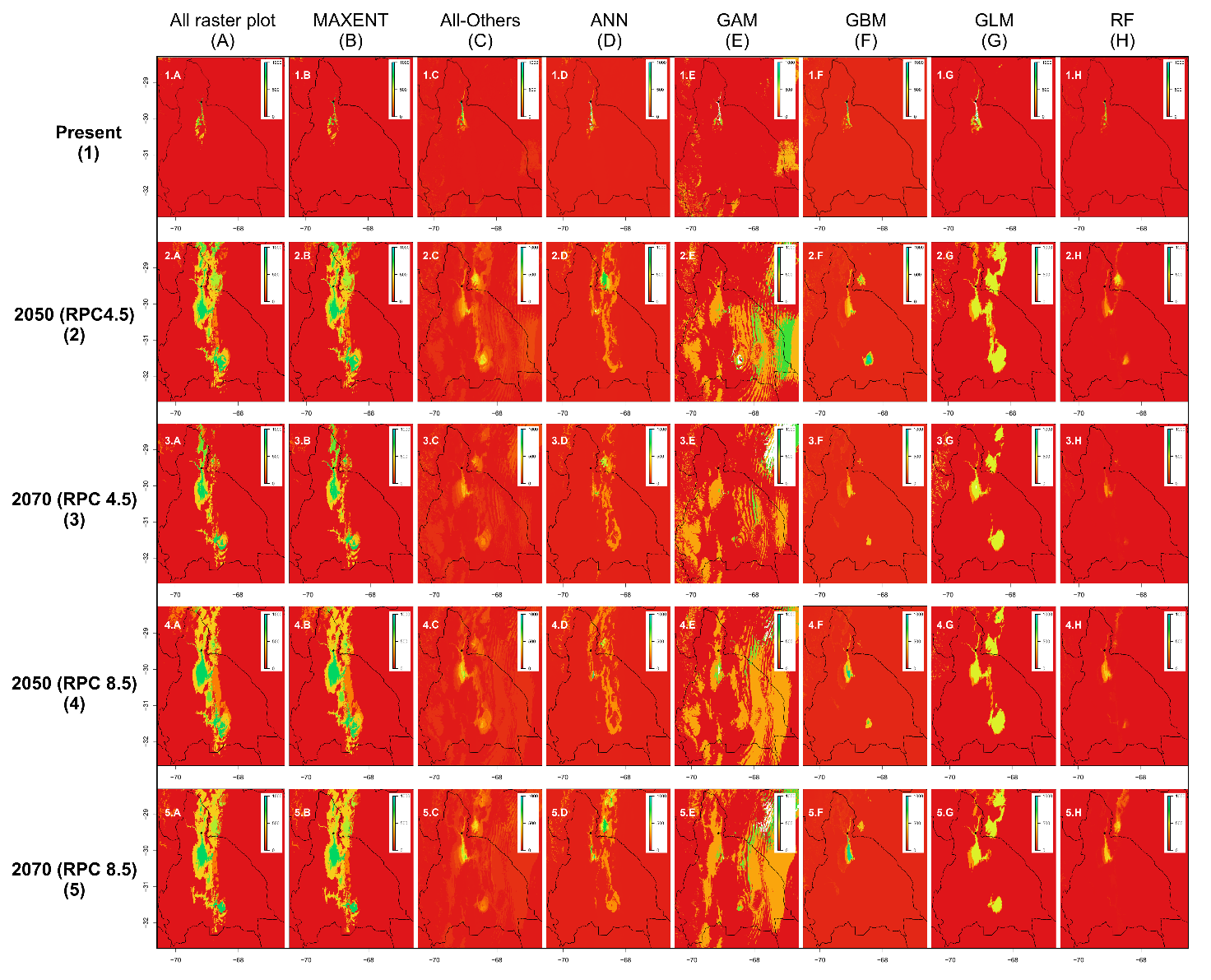


**Supplementary References**

1. Cabrera, M. R. & Monguillot, J. C. A new Andean species of Liolaemus of the darwinii complex (Reptilia: Iguanidae). *Zootaxa* **1106**, 35‒43 (2006).

2. Phillips, S. J. *et al.* Sample selection bias and presence-only distribution models: implications for background and pseudo-absence data. *Ecol. Appl.* **19**, 181–197 (2009).

3. Iturbide, M. *et al.* A framework for species distribution modelling with improved pseudo-absence generation. *Ecol. Model.* **312**, 166–174 (2015).

4. Caetano, G., Santos, J. C. & Sinervo, B. *Mapinguari: Process-Based Biogeographical Analysis. R package ver. 1.0.* (2019).

5. Fick, S. E. & Hijmans, R. J. WorldClim 2: new 1‐km spatial resolution climate surfaces for global land areas. *Int. J. Climatol.* **37**, 4302–4315 (2017).

6. Luedeling, E., Kunz, A. & Blanke, M. M. Identification of chilling and heat requirements of cherry trees—a statistical approach. *Int. J. Biometeorol.* **57**, 679–689 (2013).

7. Beckman, W. A., Mitchell, J. W. & Porter, W. P. Thermal model for prediction of a desert iguana’s daily and seasonal behavior. *J. Heat Transfer* **95**, 257–262 (1973).

8. Buckley, L., Briones-Ortiz, B., John, A., Levy, O. & Sakairi, Y. *TrenchR: An R package encompassing functions for assessing the impact of the environment of organisms. R package ver 0.0.0.9000.* (2017).

9. Gates, D. M. *Biophysical Ecology. 1st edn*. (Springer-Verlag New York, USA, 1980).

10. Oswald, S. A., Nisbet, I. C. T., Chiaradia, A. & Arnold, J. M. FlexParamCurve: R package for flexible fitting of nonlinear parametric curves. *Methods Ecol. Evol.* **3**, 1073–1077 (2012).

11. Sinervo, B. *et al.* Erosion of lizard diversity by Climate Change and altered thermal niches. *Science* **328**, 894–899 (2010).

12. Fuka, D. R., Walter, M. T., Archibald, J. A., Steenhuis, T. S. & Easton, Z. M. *EcoHydRology: A Community Modeling Foundation for Eco-Hydrology. R package ver. 0.4.12.1.* (2018).

13. Allen, R. G., Pereira, L. S., Raes, D. & Smith, M. *Crop Evapotranspiration: Guidelines for Computing Crop Water Requirements. FAO Irrigation and drainage paper N° 56*. (Food and Agriculture Organization of the United Nations, Rome, Italy, 1998).

14. Greenwell, B., Boehmke, B., Cunningham, J. & GBM developers. *GBM: Generalized Boosted Regression Models. R package ver. 2.1.8.* (2018).

15. Liaw, A. & Wiener, M. Classification and Regression by randomForest. *R News* 18‒22 (2002).

16. Naimi, B., Hamm, N. A. S., Groen, T. A., Skidmore, A. K. & Toxopeus, A. G. Where is positional uncertainty a problem for species distribution modelling? *Ecography* **37**, 191–203 (2014).

17. Thuiller, W., Georges, D., Engler, R. & Breiner, F. *biomod2: Ensemble Platform for Species Distribution Modeling. R package ver. 3.4.6.* (2020).

18. Hijmans, R. J. *raster: Geographic Data Analysis and Modeling. R package ver. 3.4-5*. (2020).

19. Hijmans, R. J., Phillips, S., Leathwick, J. & Elith, J. *dismo: Species Distribution Modeling. R package ver. 1.1-4.* (2017).

20. R Core Team. *R: A language and environment for statistical computing. R Foundation for Statistical Computing*. (2019).
